# HBOCpred: An Ensemble Framework for Prioritisation of Germline Missense Variants in Hereditary Breast and Ovarian Cancer

**DOI:** 10.3390/ijms27198865

**Published:** 2026-10-05

**Authors:** Mustafa Tarık Alay, Haktan Bağış Erdem

**Affiliations:** Department of Medical Genetics, Ankara Etlik City Hospital, 06170 Ankara, Türkiye; haktanbagis@gmail.com

**Keywords:** hereditary breast and ovarian cancer, variant of uncertain significance, missense variant, machine learning, ensemble learning, variant prioritisation

## Abstract

HBOCpred was developed to support research prioritisation of unresolved germline missense variants in a defined 17-gene study panel. Four calibrated learners provide vote states (V0–V4) and a continuous modified adaptive classifier score (S_MAC_). The same directional descriptors apply to labelled and unresolved records: unanimous benign/likely benign-directed (V0), mixed (V1–V3), and unanimous likely pathogenic/pathogenic-directed (V4) votes. Development used 2160 anchors and five repeats of nested five-fold cross-validation, with preprocessing fitted within training partitions. The annotation-completeness rule permitted evaluation of 2046 anchors per repeat. Mean balanced accuracy for the secondary ≥3-of-4 endpoint was 0.9604; mean S_MAC_ area under the receiver operating characteristic curve (AUROC) and Brier score were 0.9909 and 0.0294. In the first repeat, unanimity covered 1968/2160 anchors (91.11%), with source-label agreement in 1920/1968 unanimous outputs (97.56%). Leave-one-gene-out AUROC was 0.9812 among 2045 evaluable anchors, with substantial gene-specific variation. Among 43,679 unresolved records, V0, V1–V3, and V4 contained 33,414, 4491 and 5774 variants, respectively. HBOCpred makes learner agreement and disagreement available for expert review and functional follow-up. Independent validation is needed to assess its performance and utility in unresolved variants.

## 1. Introduction

Hereditary breast and ovarian cancer (HBOC) is an autosomal dominant cancer-predisposition syndrome primarily associated with an increased risk of breast cancer and tubo-ovarian cancer. Inherited predisposition due to pathogenic germline variants in HBOC genes is estimated to account for 5–10% of breast cancers and 10–15% of ovarian cancers [1,2]. HBOC is a genetically heterogeneous disorder, and the implicated gene influences penetrance, age at onset, tumour spectrum, and clinical presentation.

Among the principal high-penetrance breast cancer susceptibility genes, *BRCA1* and *BRCA2* are the best characterised and remain the most common genetic causes of classical HBOC. Both genes encode central components of the homologous recombination pathway involved in repairing DNA double-strand breaks. Germline pathogenic variants in both genes confer substantially increased risks of breast and tubo-ovarian cancer. By 80 years of age, the cumulative breast cancer risk is estimated at approximately 72% for *BRCA1* and 69% for *BRCA2* carriers, whereas the corresponding ovarian cancer risks are approximately 44% and 17%, respectively [3].

Accordingly, hereditary breast and ovarian cancer testing historically focused on *BRCA1* and *BRCA2*, although contemporary diagnostic practice increasingly uses multigene panels. *PALB2* is also established as a high-penetrance breast cancer susceptibility gene, with the cumulative breast cancer risk reaching approximately 50% by 80 years of age [4,5]. *TP53*, *PTEN*, *STK11*, and *CDH1* are associated with rarer high-penetrance cancer-predisposition syndromes [6], including Li–Fraumeni syndrome [7], PTEN hamartoma tumour syndrome [8], Peutz–Jeghers syndrome [9], and diffuse gastric and lobular breast cancer syndrome [10], respectively. In contrast, pathogenic variants in *ATM* and *CHEK2* generally confer moderate breast cancer risk, although the magnitude of risk may vary according to the specific variant and family history. *RAD51C* and *RAD51D* are moderate-penetrance susceptibility genes for tubo-ovarian cancer and also confer a more modest, subtype-dependent increase in breast cancer risk, particularly for oestrogen receptor-negative and triple-negative disease [11,12]. In contrast, *BARD1* is primarily a moderate-penetrance breast cancer susceptibility gene, with no established association with ovarian cancer, whereas *BRIP1* is an established tubo-ovarian cancer susceptibility gene and is not currently considered a breast cancer predisposition gene.

Next-generation sequencing-based multigene panels have broadened the diagnostic scope of hereditary cancer testing but have also increased the detection of variants of uncertain significance (VUS), most of which are missense substitutions. Expert groups such as the Evidence-based Network for the Interpretation of Germline Mutant Alleles (ENIGMA) and the Clinical Genome Resource (ClinGen) improve interpretive consistency through gene-specific integration of population, segregation, case–control, functional, and clinical evidence; however, expert curation remains resource-intensive and uneven across genes [13,14,15,16]. Under the American College of Medical Genetics and Genomics/Association for Molecular Pathology (ACMG/AMP) framework, appropriately calibrated computational predictions may contribute PP3 or BP4 evidence; however, correlated predictors should not be counted as independent lines of evidence [17,18,19]. Consequently, a substantial proportion of missense variants, particularly in non-*BRCA1/2* HBOC-associated genes, remains unresolved.

Systematic prioritisation can help direct expert review and functional follow-up, particularly where gene-specific curation frameworks remain unavailable [20]. An integrative filter using ten in silico predictors, for example, narrowed 153 eligible *BRCA1/2* VUS to 10 priority candidates [21]. Most established predictors were developed for genome-wide application and may not capture gene- or domain-specific mechanisms [22,23]. Disease-focused models offer a way to examine these constraints in HBOC-associated missense variants [24].

We developed and internally evaluated HBOCpred for research review of unresolved variants in a defined 17-gene study panel. The model combines SVM–RBF, L2 logistic regression, Extra Trees, and histogram gradient boosting. It retains calibrated learner probabilities and the complete four-bit vote vector alongside the V0–V4 agreement state and continuous S_MAC_ ranking score. An identical reporting rule is used for source-labelled anchors and unresolved application records: V0 denotes unanimous B/LB-directed votes, V1–V3 mixed votes, and V4 unanimous LP/P-directed votes. We assessed source-label consistency and annotation completeness, and then examined gene-specific performance and development-to-application shift.

## 2. Results

### 2.1. Analytic Separation and Feature Stability

Of the 90 input annotation fields, 48 representative features were selected without using variant labels. Excluding Reliability_index left 47 candidates for the primary analysis. The deployment refit retained 25 predictors, of which 22 appeared in at least 20 of the 25 outer-training panels (Table 1).

### 2.2. Fixed Learner Families and Repeated Nested Internal Evaluation

The ensemble comprised SVM–RBF, L2 logistic regression, Extra Trees, and histogram gradient boosting. Their fitted deployment vote thresholds were 0.5000, 0.6897, 0.5905, and 0.5000, respectively (Table 2).

**Figure 1:**
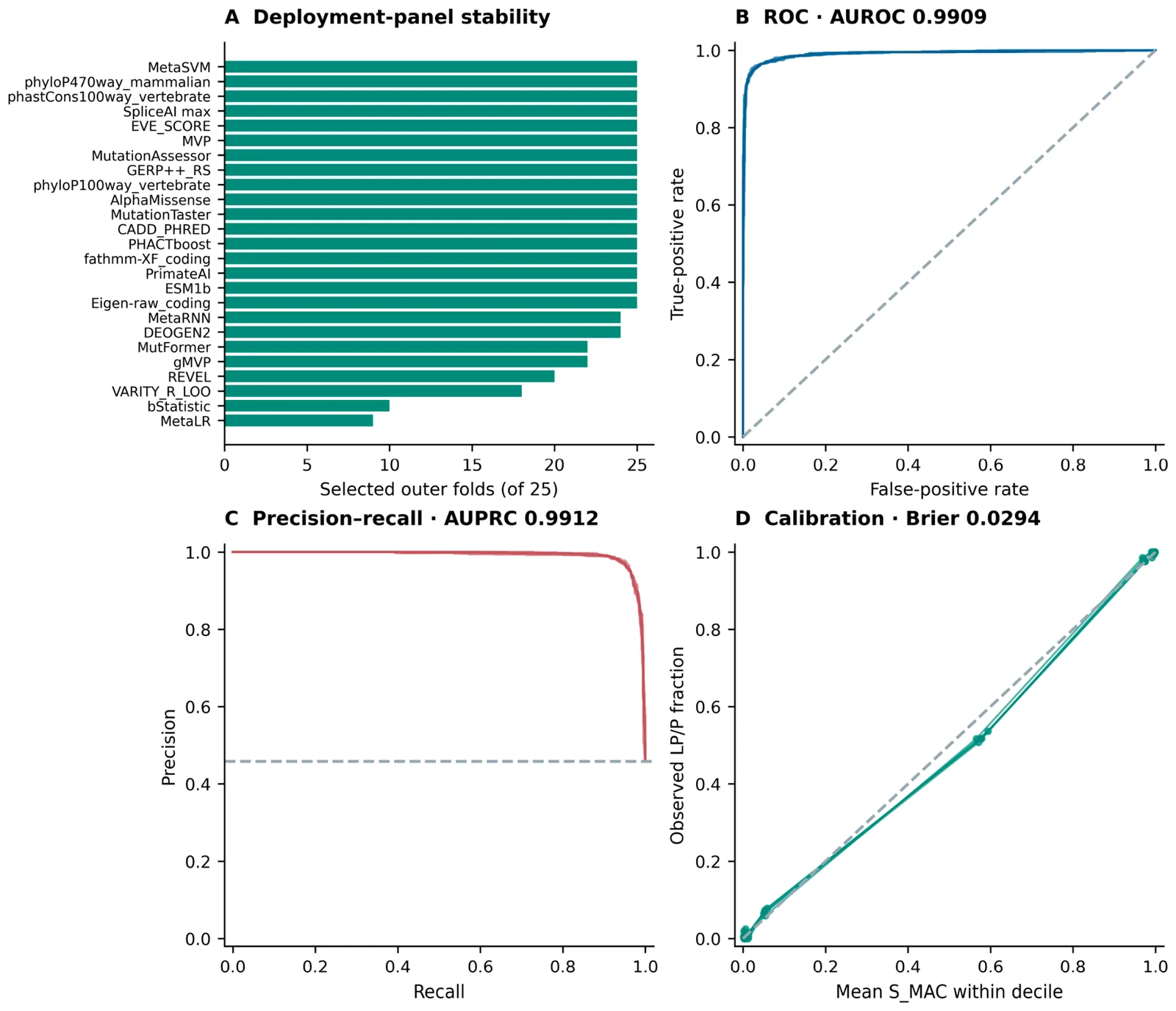
Internal evaluation of the refitted ensemble. (**A**) Selection frequency of the deployment predictors across 25 outer-training panels. (**B**) S_MAC_ ROC curves for five outer-test repeats. (**C**) Precision–recall curves; the baseline is the LP/P proportion among evaluable anchors. (**D**) Decile calibration curves. Each repeat includes 2046 evaluable anchors and excludes 114 records failing the shared annotation-completeness rule. Repeated curves show split stability within the source cohort.

Across five complete outer-test repeats, the same 2046/2160 anchors (94.72%; 1107 B/LB and 939 LP/P) satisfied the annotation-completeness rule in every repeat. Recalculation from each outer model’s selected input panel confirmed all 10,800 record-level flags, with no changes in evaluability across repeats (Supplementary Table S1). Among evaluable anchors, the secondary ≥3-of-4 endpoint achieved balanced accuracy 0.9604 ± 0.0023, sensitivity 0.9457 ± 0.0021, and specificity 0.9751 ± 0.0037. Mean PPV, NPV, F1, and MCC were 0.9699, 0.9549, 0.9576, and 0.9228. S_MAC_ yielded AUROC 0.9909 ± 0.0013, AUPRC 0.9912 ± 0.0013, and Brier score 0.0294 ± 0.0010 (Figure 1). Results are reported as mean ± sample SD across repeats.

The 114 nonevaluable anchors comprised 80 B/LB and 34 LP/P records. Their model outputs were suppressed, and they were retained in source-cohort accounting. Outer-test calibration slopes ranged from 0.968 to 1.036 and intercepts from −0.148 to −0.023.

In the first-repeat out-of-fold audit, V0 contained 1078 anchors (1046 B/LB and 32 LP/P), V1–V3 contained 78 (45 B/LB and 33 LP/P), and V4 contained 890 (16 B/LB and 874 LP/P); 114 were incomplete (Table 3). Unanimous-vote coverage was 1968/2160 (91.11%) of all source anchors, or 1968/2046 (96.19%) of evaluable anchors, with source-direction agreement in 1920/1968 unanimous outputs (97.56%). Opposite unanimous direction occurred for 32/973 LP/P anchors at V0 (3.29%; Wilson 95% interval, 2.34–4.61%) and 16/1187 B/LB anchors at V4 (1.35%; 0.83–2.18%). These proportions use all source records of the respective label; their ratio was 2.44.

### 2.3. Shared Output Descriptors and Vote-Pattern Audit

**Figure 2:**
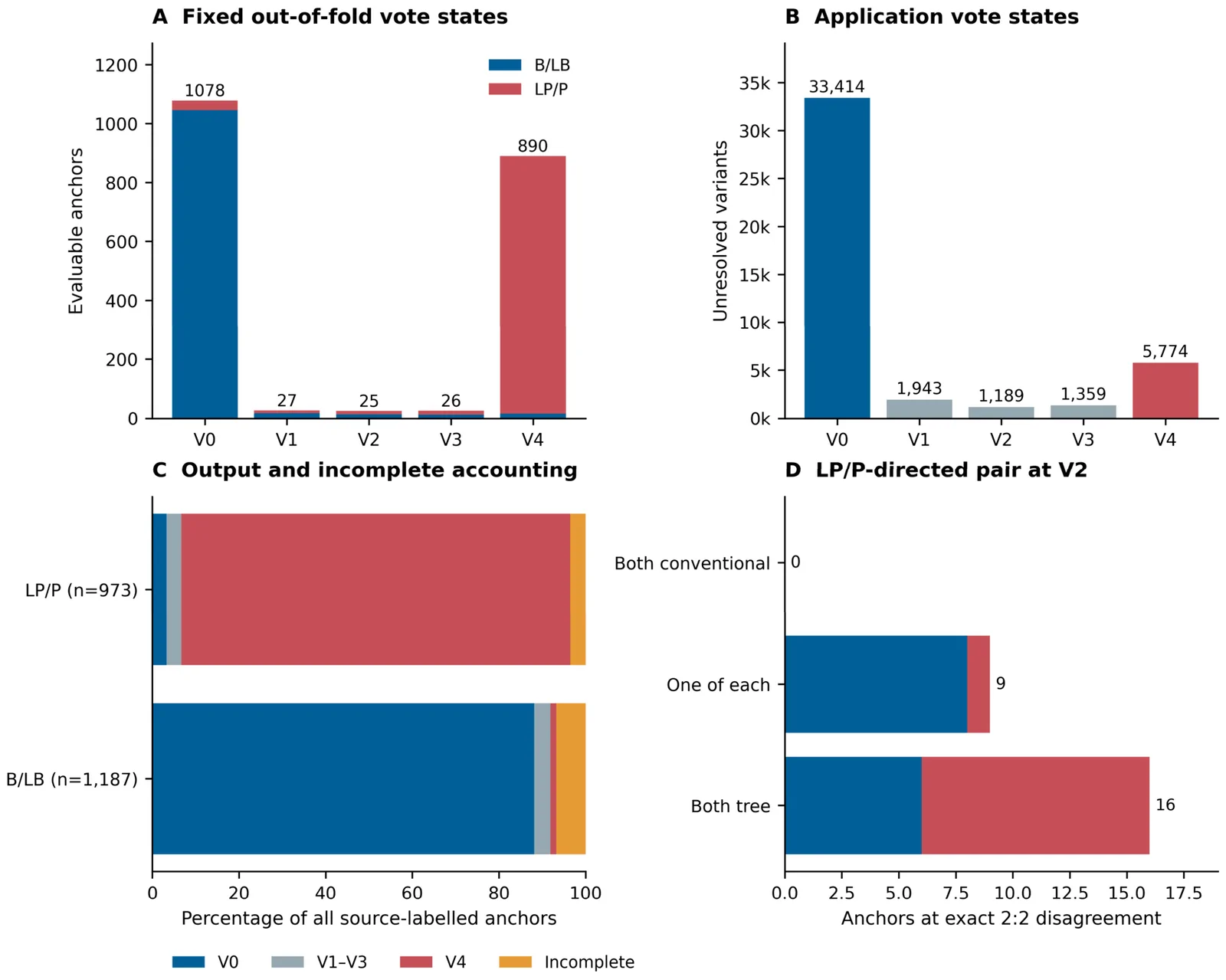
Shared vote states and completeness accounting. (**A**) First-repeat evaluable anchor counts by V-state and source label. (**B**) Deployment counts among 43,679 unresolved records. (**C**) Vote groups and incomplete records as percentages of all anchors within each source label. (**D**) Identities of the two LP/P-directed learners at V2; colours indicate source labels.

The deployment model assigned the 43,679 unresolved records to V0 (33,414), V1 (1943), V2 (1189), V3 (1359), and V4 (5774). The shared descriptors therefore comprised 33,414 unanimous B/LB-directed, 4491 mixed-vote, and 5774 unanimous LP/P-directed records (Figure 2). Exact V-states, learner identities, and probabilities remain available alongside S_MAC_.

Mixed votes occurred in 78/2046 evaluable first-repeat anchors (3.81%), compared with 4491/43,679 application records (10.28%), a 2.70-fold difference. This comparison conditions on evaluability in both datasets; the 114 incomplete anchors are reported separately.

The first-repeat mixed-vote states contained 27 V1 anchors (9 LP/P), 25 V2 anchors (11 LP/P), and 26 V3 anchors (13 LP/P). The LP/P fractions were 44.0% at V2 (Wilson 95% interval, 26.7–62.9%) and 50.0% at V3 (32.1–67.9%). Among these 78 mixed-vote anchors, the secondary ≥3-of-4 endpoint agreed with source labels in 45/78 (57.69%), with balanced accuracy 0.5525.

At exact 2:2 disagreement, the two tree learners supplied the LP/P-directed votes in 16/25 anchors (10 LP/P and 6 B/LB). One tree and one conventional learner formed the pair in 9/25 (1 LP/P and 8 B/LB); the two conventional learners never formed the pair.

### 2.4. Sensitivity Analyses

In first-repeat five-fold sensitivity analyses, including Reliability_index yielded balanced accuracy 0.9614 and AUROC 0.9891, versus 0.9592 and 0.9886 in the primary analysis. Excluding BayesDel addAF and AlphaMissense from the candidate pool before repeating feature selection and fitting yielded 0.9607 and 0.9896. Refitting on the 2120 variants with a current same-gene transcript missense annotation yielded balanced accuracy 0.9615 and AUROC 0.9902 among 2019 evaluable records; restricting the primary predictions to the same records yielded 0.9584 and 0.9884 (Table 4). Of the 1998 anchors whose embedded ClinVar significance agreed with the processed source label, 1985 (1081 B/LB and 904 LP/P) were evaluable in every repeat; 13 (2 B/LB and 11 LP/P) failed the completeness rule. Across the five repeats, mean balanced accuracy was 0.9598 ± 0.0022 and AUROC 0.9907 ± 0.0013.

### 2.5. Gene-Held-Out Evaluation and Reference-Source Audits

**Figure 3:**
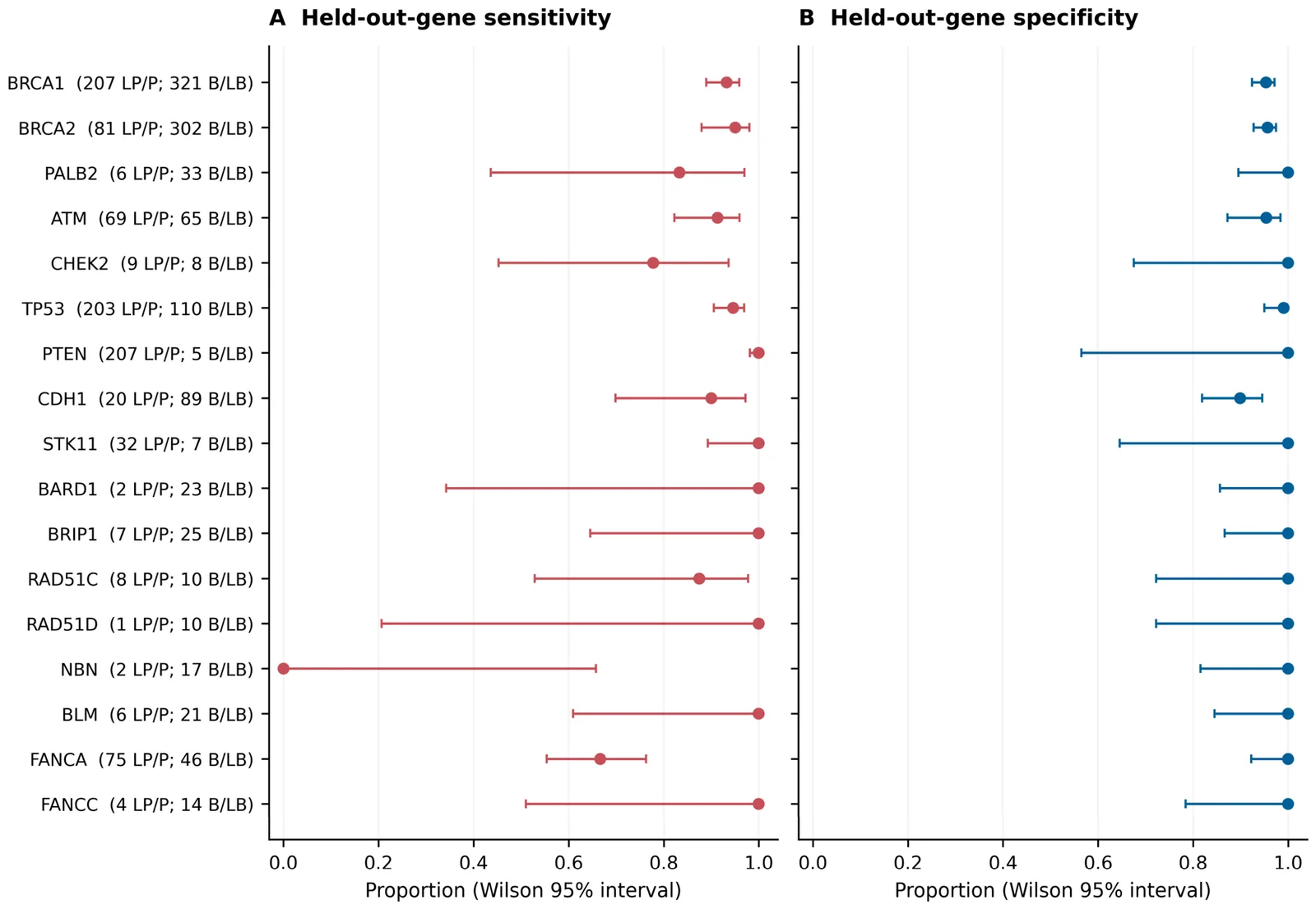
Per-gene leave-one-gene-out evaluation. (**A**) Held-out-gene sensitivity, calculated among LP/P anchors. (**B**) Held-out-gene specificity, calculated among B/LB anchors. Red points and error bars indicate sensitivity estimates and their Wilson 95% confidence intervals, whereas blue points and error bars indicate specificity estimates and their Wilson 95% confidence intervals. Row labels give the evaluable LP/P and B/LB denominators for each gene. Each gene was excluded from every fitting stage of its corresponding model. Incomplete records were excluded from these fractions and retained in the supplementary per-gene accounting.

Leave-one-gene-out evaluation across the 17 study genes included 2045 evaluable anchors; 115 anchors lacked sufficient predictor data. At the ≥3-of-4 threshold, pooled balanced accuracy was 0.9453, sensitivity 0.9276, and specificity 0.9629. SMAC had an AUROC of 0.9812, an AUPRC of 0.9810, and a Brier score of 0.0443. For *BRCA1*, sensitivity was 193/207 (93.2%) and specificity 306/321 (95.3%); the corresponding estimates for *BRCA2* were 77/81 (95.1%) and 289/302 (95.7%). Sensitivity was lower for *FANCA* (50/75, 66.7%) and *NBN* (0/2) [Figure 3]. *PTEN* specificity was 5/5, but 17 additional B/LB anchors did not meet the completeness criterion [Supplementary Table S2]. Forty-seven hospital variant records from patients with a personal history of HBOC-associated cancer and at least four affected family members were included. These records involved 8 of the 17 study genes: *ATM*, *BRCA1*, *BRCA2*, *CHEK2*, *FANCA*, *PALB2*, *RAD51C*, and *TP53*. After transcript harmonisation and deduplication, they represented 31 distinct missense variants [Table 5]. All 31 met the ≥20/25 completeness criterion and were scored using the frozen deployment model. Twenty-two had been used to fit the model, so their predictions were in-sample. Eight were in the frozen application catalogue, and one was absent from both datasets. *CHEK2* p.Ile157Thr received V0 (SMAC = 0.0093). Hospital reference labels were not independently adjudicated, and no external performance estimates were calculated.

The gnomAD eligibility audit identified 50 missense candidates: 45 overlapped the 2160 development anchors, and one overlapped the 43,679-record application catalogue. Four were absent from both study datasets. *TP53* c.215C>G p.(Pro72Arg) had 24/25 predictors and received V0 (0000; S_MAC_ = 0.0007) under the unchanged frozen deployment model. *BRCA2* c.10234A>G p.(Ile3412Val) had only 1/25 predictors; the *CHEK2* and *PTEN* candidates were missense on alternative transcripts but not on MANE Select and lacked matched numeric inputs. These three candidates were not scored. No benign-set performance estimate was calculated (Table S3).

### 2.6. Constituent-Score Comparisons and Review-Status Results

**Figure 4:**
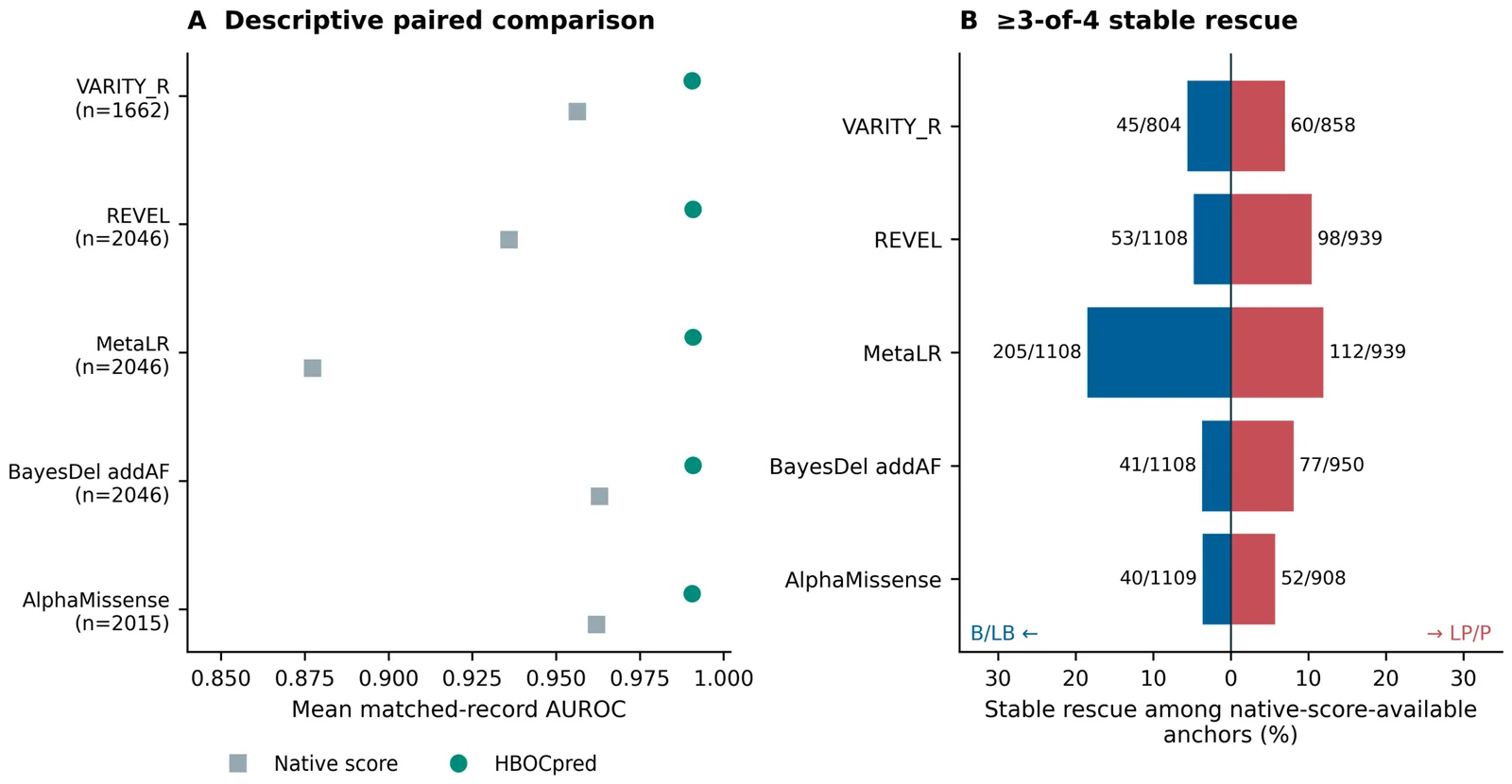
Descriptive constituent-score comparisons. (**A**) Mean AUROC on matched records with an observed comparator score and an evaluable HBOCpred output; sample sizes are shown. (**B**) Stable rescue at the secondary ≥3-of-4 endpoint: correct ensemble direction and incorrect comparator direction in all five outer-test appearances. Within each source-label group, denominators include all anchors with an observed native comparator score, including incomplete records, and can exceed the evaluable denominators in panel (**A**).

Matched-record comparisons included REVEL, MetaLR, VARITY_R, BayesDel addAF, and AlphaMissense. Across repeats, S_MAC_ AUROC was approximately 0.9906–0.9909 on the respective matched subsets, compared with 0.8773–0.9630 for the native scores (Figure 4A). For the secondary ≥3-of-4 endpoint, stable rescue in all five outer-test appearances was observed for 53/1108 B/LB and 98/939 LP/P source anchors relative to REVEL, and 205/1108 B/LB and 112/939 LP/P anchors relative to MetaLR (Figure 4B). These denominators comprise all source anchors with an observed native comparator score within each label group, including incomplete records.

**Figure 5:**
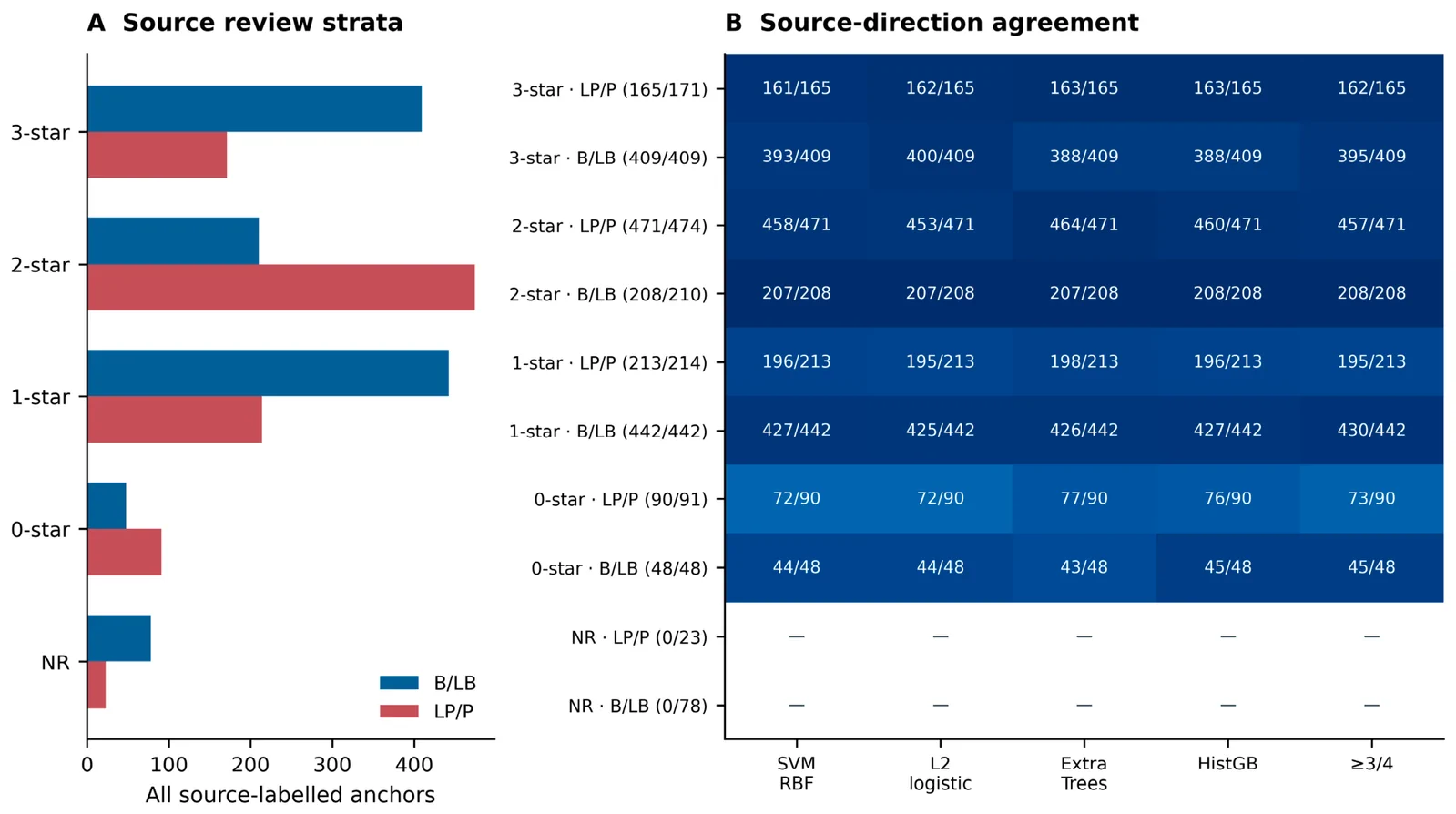
Review-status accounting and source-label agreement. (**A**) Source counts by recorded review status. (**B**) Correct/evaluable counts for each learner and the secondary ensemble endpoint in the first repeat. A dash denotes no evaluable denominator.

The review-status audit separates source counts from evaluable counts (Figure 5). The NR category contained 101 records, comprising 78 B/LB and 23 LP/P, all of which failed the completeness rule. Consequently, no learner-concordance fraction was estimable for NR. Results for recorded review-status strata are shown with their evaluable denominators.

### 2.7. Distribution Shift, Gene Signal, and Application Catalogue

**Figure 6:**
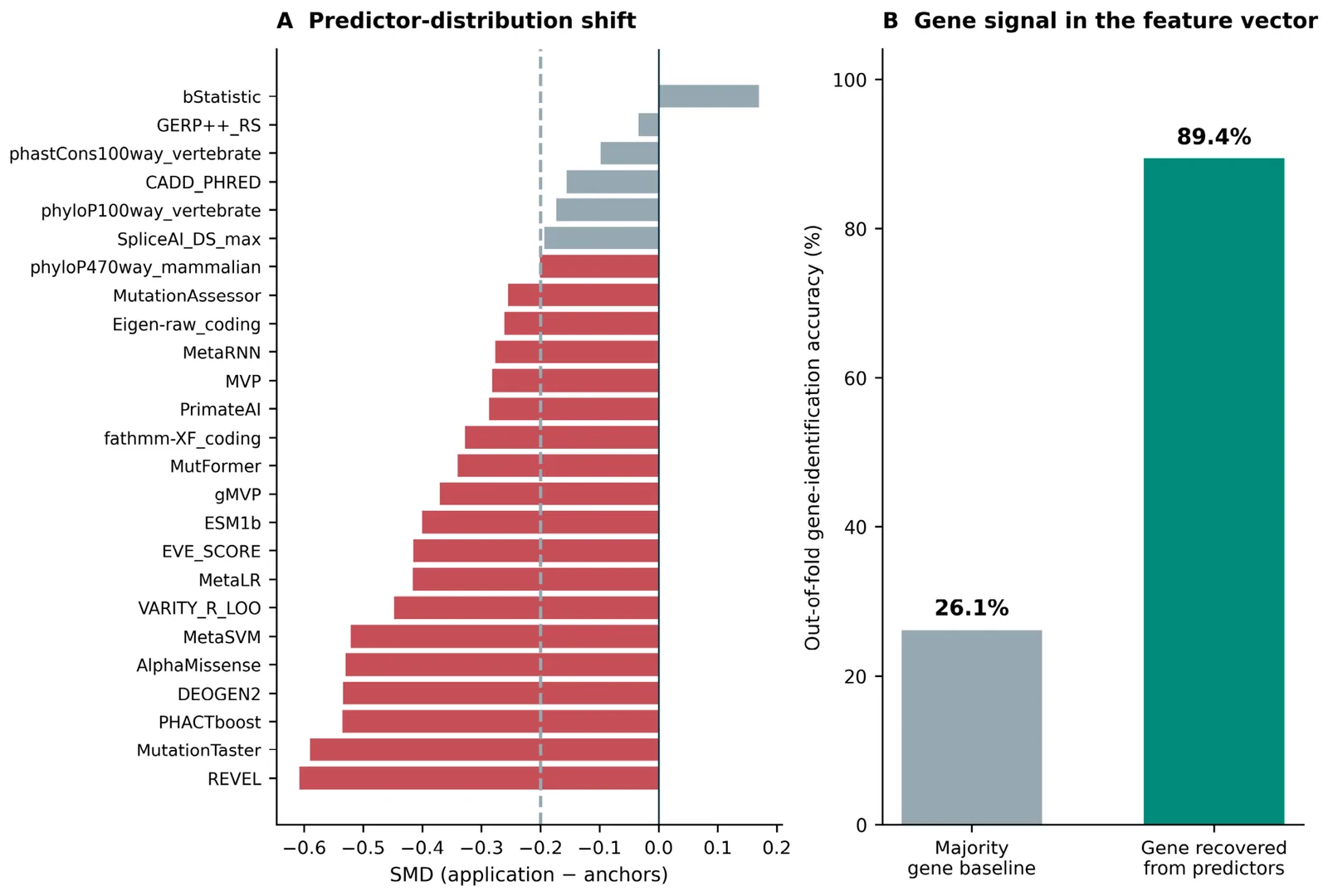
Development-to-application shift and gene information. (**A**) Standardised mean differences (SMDs) for the 25 deployment predictors. Negative values indicate lower observed values in the application catalogue. Red bars indicate predictors with SMD < −0.20, whereas grey bars indicate predictors with SMD ≥ −0.20; the dashed line marks the −0.20 threshold. (**B**) First-repeat out-of-fold gene-identification accuracy from training-selected predictors versus a majority-gene baseline. Grey indicates the majority-gene baseline, and green indicates gene identification from the predictor set.

Nineteen of the 25 deployment predictors had absolute standardised mean differences of at least 0.20 between anchors and the application catalogue; 24 shifted in the negative direction (Figure 6A). An auxiliary classifier recovered gene identity from outer-training-selected predictors with 89.40% out-of-fold accuracy, compared with 26.11% for the majority-gene baseline (Figure 6B). A separate gene-prevalence-only classifier yielded source-label AUROC 0.7553. A descriptive reanalysis of the saved primary out-of-fold scores gave a mean within-gene AUROC of 0.9877 ± 0.0016 when weighted by the number of same-gene LP/P–B/LB pairs, and an unweighted gene mean of 0.9734 ± 0.0103 across the five repeats. All 17 genes had both evaluable source-label classes. Gene-specific AUROCs are reported in Supplementary Table S2 and Supplementary Data S1, with primary cross-validation and gene-held-out results identified separately.

All 43,679 application records satisfied the deployment requirement of at least 20 observed predictors out of 25; the minimum was 20 and the median 24. The frozen catalogue includes variant identifiers, gene labels, individual learner probabilities, vote vectors and V-states, directional descriptors, S_MAC_, and completeness information. Source classification is retained separately from the model descriptor.

**Figure 7:**
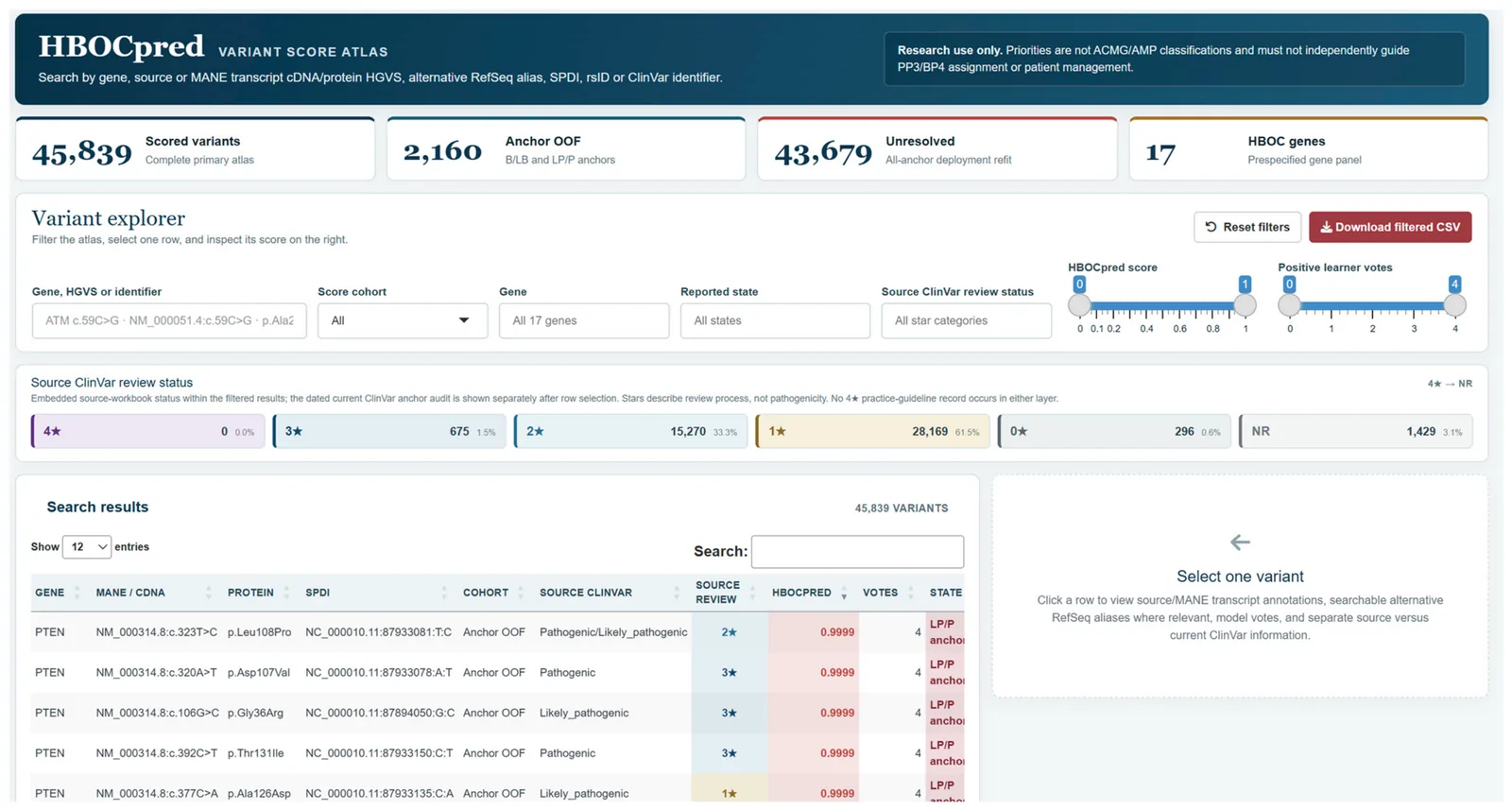
Companion Shiny variant catalogue. The screenshot shows the HBOCpred viewer with all 43,679 application records and shared vote-group counts. Available at: https://alaymd.shinyapps.io/hbocpred/ (accessed on 15 September 2026).

The companion Shiny catalogue viewer supports gene and variant searches, displays individual learner scores and vote patterns, and exports filtered records (Figure 7). The viewer is available at https://alaymd.shinyapps.io/hbocpred/ (accessed on 15 September 2026); its source and complete 43,679-record application catalogue are included in the reproducibility package.

## 3. Discussion

We developed HBOCpred to support research prioritisation of germline missense variants in a defined 17-gene panel. SVM–RBF, L2 logistic regression, Extra Trees, and histogram gradient boosting provide individual probabilities and votes alongside the modified adaptive classifier score (S_MAC_). The same directional descriptors apply to labelled anchors and unresolved variants. Across five repeats, the secondary ≥3-of-4 endpoint gave balanced accuracy 0.9604 ± 0.0023 among 2046 evaluable anchors out of 2160. For the fixed vote-state audit, we report repeat 1: unanimity covered 1968/2160 anchors (91.11% of the full cohort), and source-label agreement was 1920/1968 (97.56%), conditional on unanimity.

Four learners can produce six distinct two-vote combinations. In the first-repeat audit, the two tree learners supplied the LP/P-directed pair in 16 of the 25 V2 anchors; in the remaining 9 anchors, the pair combined one tree and one non-tree learner. We retain learner identity so that individual decisions can be inspected within the same vote count. Whether this information improves prioritisation beyond S_MAC_ alone remains to be tested.

Earlier disease-focused studies selected boosting or random-forest models for *BRCA1/2* [25,26,27], and Ahmad et al. selected Extra Trees after comparing nine algorithms in breast-cancer-associated genes [28]. Broader hereditary-cancer tools include HCSpred, first presented in 2025 [29], and the random-forest-based HerCanPred [24]. These studies provide methodological context, although differences in variant selection and evaluation design prevent direct performance comparisons.

Lee et al. [30] offer a closer comparison. They combined autoencoder pretraining with 50 independently fine-tuned DNNs of a shared architecture, averaged the predictions and examined feature contributions for individual variants. Meta-EA learns gene-specific weights for existing predictors using Evolutionary Action as the reference [31]. Katsonis and Lichtarge also found that predictor scores agreed more closely with one another than with experimental measurements, particularly for *CALM1* and pyruvate kinase [31]. In HBOCpred, we retain the thresholded decisions of four distinct algorithms as well as their aggregate score, so reviewers can see which learners support each direction.

Mean4 is the arithmetic mean of the four calibrated learner probabilities. S_MAC_ is a logistic recalibration of logit(Mean4), providing a continuous ordering within vote states [22,32]. In the deployment fit, this transformation is monotone with a positive slope, so S_MAC_ preserves the Mean4 ranking while changing the probability scale. Calibration against source labels does not establish clinical posterior probabilities or PP3/BP4 evidence thresholds [19,33].

Performance varied between genes. Gene identity was recoverable from the predictors with 89.40% out-of-fold accuracy, and a gene-prevalence-only model achieved AUROC 0.7553. In a descriptive analysis of the saved predictions, the pair-weighted mean within-gene AUROC was 0.9877 ± 0.0016 across repeats. Thus, discrimination remained high when comparisons were confined to the same gene, although this does not isolate the sources of within-gene signal or remove upstream label dependence. In the separate gene-held-out analysis, *FANCA* sensitivity was 50/75 at the ≥3-of-4 endpoint; this concerns thresholded calls among evaluable LP/P anchors, rather than agreement conditional on unanimity. *NBN* sensitivity and *PTEN* specificity rested on only two and five evaluable anchors. Chow et al. [34] likewise reported gene-dependent associations with breast cancer risk and limited utility in VUS-only analyses. The gene-specific criteria used for Alport syndrome [35] and the regional modelling of cardiomyopathy variants [36] illustrate why pooled performance should be accompanied by gene-level results.

The mean internal S_MAC_ AUROC of 0.9909 needs to be read in the context of the reference labels. Wilcox et al. showed that computational evidence can contribute to clinical variant classifications [18], and several HBOCpred inputs originate from predictors trained on clinical labels. Source classifications may also incorporate PP3/BP4 evidence from related predictors. Holding a variant out of our model therefore leaves possible links to upstream training and label formation. We excluded BayesDel addAF and AlphaMissense in a sensitivity analysis and observed little change in discrimination. Other clinically trained or frequency-informed predictors remained, so this result leaves circularity unquantified.

Mixed votes were 2.70 times as frequent in the unresolved catalogue as in first-repeat out-of-fold anchor outputs, alongside differences in the observed predictor distributions. The two sets were also scored by different fits: outer-training models for anchors and the full-cohort deployment model for catalogue records. Selection through the availability of decisive classification evidence may contribute to the difference, as may gene composition and fitted thresholds. The observed ratio therefore combines population and model effects that this analysis cannot separate. Internal source-label estimates may therefore overstate performance in unresolved variants; the magnitude of any such optimism cannot be estimated from these data.

Even unanimous votes occasionally opposed the source label. V0 contained 32 of the 973 source LP/P anchors (3.29%), and V4 contained 16 of the 1187 source B/LB anchors (1.35%). The ratio of these source-cohort rates, (32/973)/(16/1187), was 2.44; it does not measure relative clinical harm. A V0 result cannot justify excluding a variant from review, and a V4 result is insufficient for a pathogenic clinical classification.

The 43,679-record catalogue makes these decisions available for review, together with learner probabilities and annotation completeness. Nakahara et al. [21] linked computational prioritisation of *BRCA1/2* variants detected by tumour genomic profiling with functional investigation; Zanti et al. [14] demonstrated the contribution of case–control evidence to *BRCA1/2* classification. Such evidence provides a way to assess variants selected from the catalogue. Future studies could compare review based on S_MAC_ alone with review that also includes the learner vote pattern, measuring the yield of independently supported variants.

### 3.1. Limitations

We evaluated a retrospectively assembled cohort with unequal gene representation and source-label prevalence. The learner families and reporting convention were chosen during earlier development. Nested resampling evaluates the resulting pipeline but does not capture uncertainty from those choices. The five-repeat SDs describe split stability; conditional first-repeat intervals omit uncertainty from model refitting and gene clustering. The small V2 and V3 strata provide no basis for an ordinal clinical scale, and learners sharing inputs cannot be treated as independent evidence.

Of the 2160 total anchors, 2046 met the annotation-completeness rule, and 114 failed it in every repeat. The incomplete group included all 101 NR records, leaving no evaluable NR stratum. A total of 40 anchors lacked a current same-gene missense annotation: 13 were among the 114 incomplete records, and 27 were evaluable under the numeric completeness rule. Historical transcript assignments remain incompletely documented despite the transcript-restricted analysis. Distribution differences further restrict transfer of internal estimates to unresolved variants.

The hospital and gnomAD audits yielded no independent clinical performance estimate. Most variants overlapped the study cohorts, hospital labels were not independently adjudicated, and only one additional gnomAD candidate had sufficient inputs for scoring. The model is restricted to germline missense variants in the 17-gene panel and does not incorporate patient-level clinical evidence. Balanced accuracy weights the two source-label classes equally; the clinical consequences of missed and unnecessary follow-up require separate assessment.

### 3.2. Conclusions

HBOCpred provides an auditable framework for research prioritisation of unresolved germline missense variants within a defined 17-gene panel. Shared V0–V4 descriptors report model direction and agreement, while individual learner probabilities and the full vote pattern remain available alongside S_MAC_. Internal evaluation showed high source-label discrimination among evaluable anchors, with gene-specific and annotation-related limitations. The catalogue supports expert review and research follow-up. Its value for prioritising unresolved variants, including any benefit from displaying the vote pattern, requires independent evaluation.

## 4. Materials and Methods

### 4.1. Study Design

**Figure 8:**
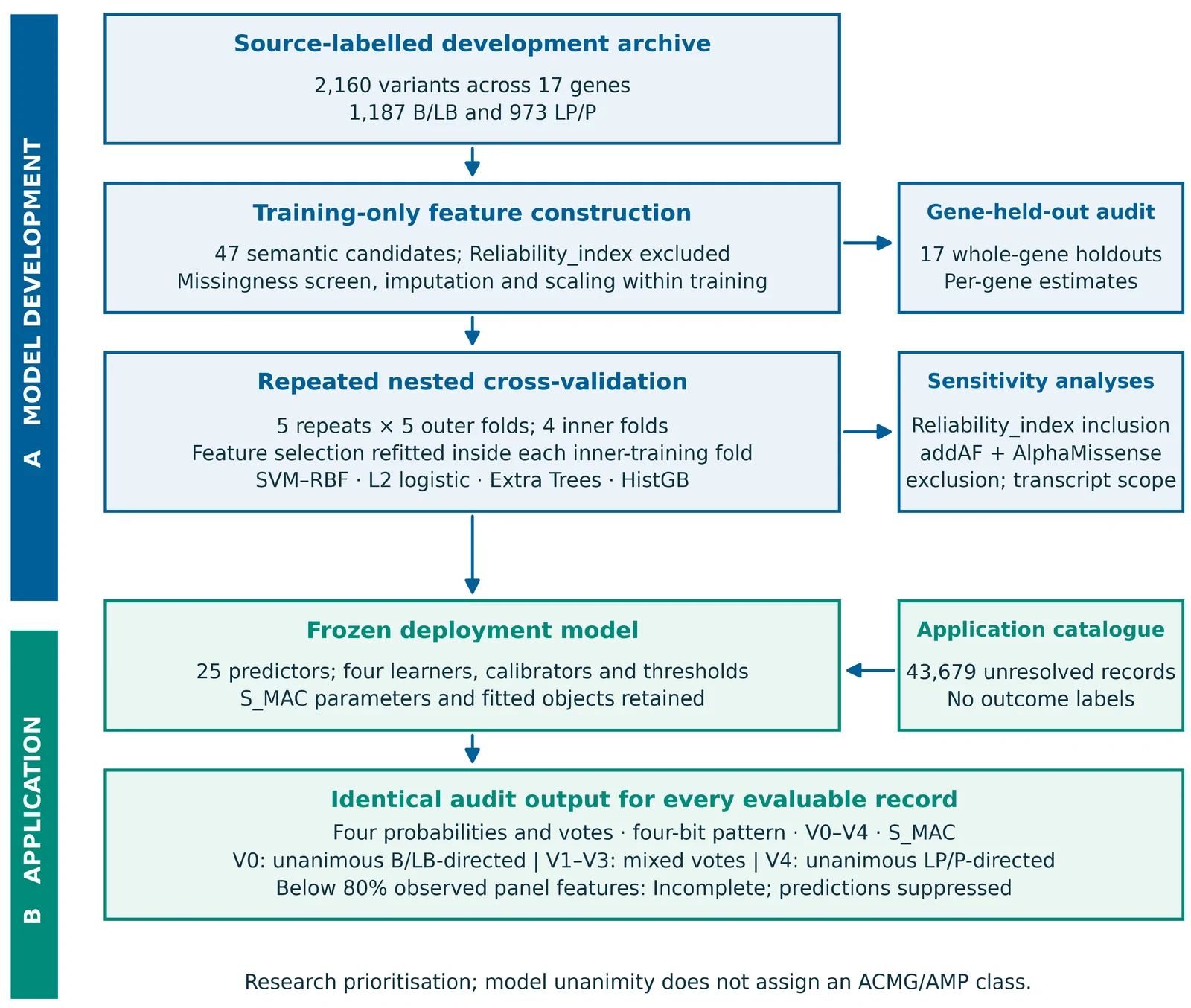
Development and shared reporting workflow. The source-labelled archive contains 2160 anchors across 17 genes. Label-independent numeric processing produces 47 primary candidates after excluding Reliability_index. Missingness filtering, imputation, scaling, feature selection, and tuning are refitted within each training partition. Evaluation comprises repeated nested cross-validation, complete gene holdouts and paired sensitivity analyses. A separate deployment fit uses all anchors and scores the 43,679 unresolved records without refitting. Both evaluation and application use the same directional descriptors and completeness guard.

This retrospective variant-level study develops and internally evaluates a research prioritisation model using 2160 processed source-labelled anchors, and then it applies the frozen deployment fit to a disjoint catalogue of 43,679 unresolved variants. The 17-gene study panel includes established susceptibility and syndromic genes alongside an exploratory scope that includes *FANCA*; panel membership does not itself establish monoallelic HBOC risk. The anchor cohort was originally ascertained as missense; current transcript discrepancies were retained in the primary cohort and examined through a restricted refit. No prospectively collected, independently adjudicated clinical validation cohort was analysed. All 2160 anchors were retained in source-cohort accounting without a formal sample-size calculation. The analysis was not prospectively registered, and learner-family selection and the reporting convention were informed by earlier development and revision. No resampling or class-weight correction was applied to alter class prevalence. Figure 8 summarises model development and the shared reporting workflow.

### 4.2. HBOCpred Algorithm

#### 4.2.1. Fixed Learner Families

Throughout, LP/P is coded as 1 and B/LB as 0.

The ensemble contains SVM–RBF and L2 logistic regression as conventional non-tree learners, Extra Trees as a bagging learner, and histogram gradient boosting as a boosting learner. Learner families were selected during earlier development and held fixed throughout this evaluation. Hyperparameter tuning was repeated within each training partition.

#### 4.2.2. Calibrated Adaptive Voting

For variant i and learner m, let z_im_ denote the raw SVM decision score or the clipped logit probability from a probability-output learner. A Platt transformation gives p_im_ = σ(a_m_ + b_m_z_im_), where σ(z) = 1/[1 + exp(−z)]. The learner-specific coefficients a_m_ and b_m_ are fitted using inner out-of-fold scores from the selected configuration.

(1) $$p_{\mathrm{im}}=\sigma (a_{m}+b_{m}z_{\mathrm{im}}),\;\sigma (z)=\frac{1}{1+\exp(-z)}$$

For each selected configuration, Platt calibration is cross-fitted across the four inner folds. The vote threshold maximises balanced accuracy on these cross-calibrated inner out-of-fold probabilities. A tie is resolved by the threshold closest to 0.5 and then by the lower threshold. The final calibrator is refitted on all inner out-of-fold scores, and the base learner is refitted on the complete corresponding training partition.

(2) $$\tau_{m}={\arg\;\max}_{t}\frac{{\mathrm{TPR}}_{m}(t)+{\mathrm{TNR}}_{m}(t)}{2}$$

A learner casts an LP/P-directed vote when its calibrated probability is at least its fitted threshold. In the equations, M denotes the fixed set of four learners and c_i is their total vote count.

(3) $$v_{\mathrm{im}}=I(p_{\mathrm{im}}\geq \tau_{m}),\;c_{i}=\sum_{m\in M}v_{\mathrm{im}},\;|M|=4.$$

The audit state is Vc, where c is the sum of the four LP/P-directed votes and c ∈ {0,1,2,3,4}. V2 denotes exact 2:2 disagreement. The same mapping applies to source-labelled anchors and unresolved records: V0 denotes unanimous B/LB-directed votes, V1–V3 mixed votes and V4 unanimous LP/P-directed votes. These descriptors concern model direction and agreement; they do not establish ACMG/AMP classifications, PP3/BP4 evidence strengths, clinical posterior probabilities, or an ordinal clinical scale. The secondary binary endpoint c ≥ 3 is used to calculate conventional source-label metrics alongside the reported vote states.

The complete four-bit vote vector is retained so that different learner-family configurations remain distinguishable within the same vote count.

#### 4.2.3. Continuous HBOCpred Score

Mean4 is the arithmetic mean of the four calibrated learner probabilities. The continuous modified adaptive classifier score is S_MAC_ = logistic(α + β × logit(Mean4)); α = 0.03387595 and β = 1.13536398 in the deployment fit. Meta-calibration is fitted to the mean of the inner cross-calibrated probabilities using training labels. S_MAC_ provides a continuous ranking without adding a vote or changing the directional descriptor.

#### 4.2.4. Primary Output and Completeness Flag

The output tuple includes probabilities, votes, V-state, vote vector, directional descriptor, Mean4, S_MAC_, observed-panel count, and completeness. At prediction, a record must have at least ceil(0.8 × panel size) observed predictors from the corresponding fitted model’s selected panel before imputation; the deployment panel therefore requires 20 of 25. Otherwise, all model outputs are suppressed. This rule is applied separately to each outer-test model, gene-held-out model and the deployment model. Incomplete records remain in cohort counts but are excluded from evaluable-performance denominators.

#### 4.2.5. Variant Curation and Label Definition

The study scope comprises *ATM*, *BARD1*, *BLM*, *BRCA1*, *BRCA2*, *BRIP1*, *CDH1*, *CHEK2*, *FANCA*, *FANCC*, *NBN*, *PALB2*, *PTEN*, *RAD51C*, *RAD51D*, *STK11*, and *TP53*. Model fitting and internal assessment used 2160 unique source-labelled anchors (1187 B/LB and 973 LP/P; Table 6), represented by normalised genomic identities. Historical transcript-specific inclusion decisions cannot be fully reconstructed because the retained annotations do not preserve the original consequence/transcript pairing. A current Ensembl audit found a same-gene missense annotation for 2120 anchors: 2039 on MANE Select and 81 only on other transcripts; 40 lacked such an annotation. Multi-nucleotide substitutions were retained where present. Original binary source labels were preserved, with embedded ClinVar significance and review status retained separately; 1998 anchors matched the embedded significance. NR denotes an unrecorded review-status annotation. The application catalogue contains 43,679 unresolved records with no anchor-identity overlap.

### 4.3. Feature Matrix Construction and Preprocessing

Numeric processing excluded identities, labels, categorical predictions, and annotation-position fields. The four SpliceAI delta scores [37] were collapsed to their row-wise maximum. This construction yielded 90 numeric fields; deterministic preference among semantically paired score/rank fields gave 48 candidate representatives. Reliability_index, which describes the completeness of component scores used by MetaSVM and MetaLR, was excluded from the primary analysis, leaving 47 candidates. This candidate pool is distinct from the fitted deployment panel in Table 1. No missingness screen used the unresolved application archive. Within each inner-training and outer-training partition, candidates with more than 40% missingness or no observed variation were removed; remaining missing entries were median-imputed, and values were standardised using that partition only. Four criteria were averaged by rank: orientation-independent univariate AUROC, mutual information, median absolute L1-logistic coefficient over 12 bootstrap fits with selection frequency as a tie-break, and Extra Trees importance relative to permuted shadow features over six fits. Predictors were taken greedily in consensus order, skipping candidates with absolute Pearson correlation above 0.90 with an already retained predictor, up to 25. Parameters and panels were applied unchanged to the relevant assessment records. The first finite numeric token was retained from multi-valued fields; this deterministic parsing rule does not guarantee transcript alignment.

### 4.4. Learner Specifications and Tuning

Within each training partition, six unique configurations per fixed learner family were sampled without replacement from the listed grids (Table 7); the L2 logistic grid has exactly six configurations. Configurations were ranked by mean inner-fold raw-score AUROC, then lower sample SD and finally the sorted parameter string. The algorithm key ENET_LR denotes L2 logistic regression (l1_ratio = 0). Full settings, sampled configurations, and fitted objects are included in the reproducibility archive.

### 4.5. Repeated Nested Cross-Validation

Primary evaluation used five repeats of five-fold outer cross-validation, with four-fold inner cross-validation for tuning. Gene/outcome strata with sufficient counts were retained; sparse strata were pooled by outcome. Each outer fold contained 432 source anchors. Every inner-training partition independently fitted its missingness screen, imputer, scaler, feature ranking, and correlation pruning before application to its inner assessment fold. Outer-training preprocessing was then refitted for the selected configurations. The outer-test data were excluded from every fitting step. Tuning, calibration, and threshold development used labels within outer training. Because learner families were chosen during earlier development, the outer estimates assess the specified pipeline on this source cohort without repeating the full historical model-selection process.

### 4.6. Calibration, Adaptive Voting, and Deployment Refit

Raw SVM decision values were used directly for Platt fitting. Other learner probabilities were clipped to [10^−6^, 1 − 10^−6^] and logit-transformed. Cross-calibrated inner probabilities were used for threshold development and S_MAC_ fitting, followed by complete training-partition refits. The deployment model was fitted separately using all 2160 anchors and frozen before scoring the unresolved catalogue. All fitted preprocessors, base learners, calibrators, thresholds, training identities, and inner fold memberships are retained. Deployment predictions on the anchors are explicitly marked in-sample and are not used for reported performance.

The primary descriptive audit retains exact V-states and the three shared vote groups, with incomplete accounting alongside them. Unanimity coverage is (nV0 + nV4)/n_source; conditional source-direction agreement is (nB/LB,V0 + nLP/P,V4)/(nV0 + nV4). Mixed-vote proportions used to compare populations condition on evaluability in each population. The first outer-test repeat supplies one prediction per anchor for the fixed-state tables; the performance summaries use all five repeats. Conventional binary metrics use c ≥ 3 among evaluable records, while AUROC, AUPRC, and Brier score use S_MAC_.

### 4.7. Gene-Held-Out and Sensitivity Analyses

Leave-one-gene-out evaluation comprised 17 fits, each excluding one whole gene from all training operations. Performance and evaluability were reported both pooled and separately for each held-out gene, with confusion counts and Wilson intervals. First-repeat five-fold sensitivity refits (i) included Reliability_index, (ii) excluded BayesDel_addAF_rankscore and am_pathogenicity from the candidate pool in addition to Reliability_index, and (iii) restricted training and assessment to the 2120 current same-gene-transcript missense anchors. Candidate exclusions preceded training-partition feature selection, which was repeated for each refit. BayesDel addAF was eligible in the primary candidate pool but was not retained in the final deployment panel. For the transcript-restricted analysis, primary predictions were also restricted to the same records to distinguish population restriction from refitting. Restricted models reused the original fold assignments after filtering. A separate descriptive subset analysis used primary predictions for records whose embedded ClinVar significance agreed with the processed source label.

To quantify gene information, an auxiliary 300-tree Extra Trees classifier predicted gene identity using the primary first-repeat training-selected predictor panels and training-fitted preprocessing; assessment gene labels were held out. A majority-gene classifier provided its baseline. A separate source-label baseline used only the gene-specific LP/P prevalence in the corresponding outer-training fold.

As a descriptive reanalysis of the saved primary out-of-fold predictions, we calculated S_MAC_ AUROC separately within each gene and repeat among evaluable anchors. Gene-specific means and sample SDs use the five repeats. Within each repeat, we also calculated an unweighted mean across genes and a mean weighted by nLP/P × nB/LB, the number of positive–negative pairs within each gene. These summaries compare source-label classes within genes without model refitting; they do not estimate the causal contribution of gene identity or eliminate dependence on upstream predictors. Gene-held-out AUROCs were tabulated separately because they use different fitted models.

### 4.8. Reference-Source and Application Audits

The gnomAD v4 GRCh38 query covered all 17 genes and screened PASS gene-level missense SNPs for alternate-allele frequency above 0.05 in a continental population with at least 2000 observed alleles in the same callset. Candidate identities, allele counts, denominators, and same-gene/MANE Select consequences were retained. The 50 candidates were matched by genomic allele against the 2160 development anchors and 43,679 application records; multinucleotide alleles were retained as distinct variants. Candidates absent from both datasets underwent transcript review and assessment of available numeric inputs. Source annotations were parsed with the frozen preprocessing rule, and the unchanged deployment model was applied only when at least 20 of its 25 predictors were observed. The additional TP53 record was source-labelled VUS but had an embedded ClinVar Benign annotation; absence from HBOCpred training did not establish independence from upstream predictors or clinical resources. The screen did not assign BA1 classifications or yield an independently adjudicated benign test set.

All hospital source rows were audited. In-scope variants were resolved using concordant gene, coding-allele and protein descriptions, with the supplied RefSeq transcript used where needed. Ensembl transcript snapshots and frozen catalogue annotations documented aliases. CHEK2 Ile200Thr/Ile157Thr and Gly210Arg/Gly167Arg aliases were collapsed; other CHEK2 descriptions were harmonised to MANE Select. ATM Asp2016Gly source rows contained discrepant rsIDs; matching used concordant coding/protein descriptions, while original strings and rsIDs were retained in the row-level audit. Each resolved genomic allele was scored once with the frozen deployment model and existing numeric features, subject to ≥20/25 completeness. Fitted-anchor overlap, application-catalogue overlap, and additional records outside both study datasets were recorded separately. Repeated rows were not treated as independent patients. No new model fitting or external outcome adjudication was performed.

Application-versus-anchor standardised mean differences were calculated from observed values as the application mean minus the anchor mean, divided by the square root of the mean of the two sample variances. Missingness fractions are supplied separately. Application scoring used the frozen deployment fit and the same output-completeness rule as internal evaluation. No application reference outcomes were available. Analytical figures were generated from row-level data and saved model outputs; Figure 7 is a capture of the locally running Shiny viewer.

### 4.9. Statistical Analysis

AUROC and AUPRC quantify source-label discrimination, and Brier score quantifies squared error against the binary source label; none is interpreted as clinical accuracy. Five-repeat mean ± sample SD describes split stability. The repeated predictions are not pooled as independent observations to form confidence intervals. Supplementary Table S4 gives conditional first-repeat intervals from 2000 class-stratified bootstrap resamples of one fixed out-of-fold prediction per evaluable variant (seed 20260905); these do not refit models or resample gene clusters and therefore omit training and clustering uncertainty. Wilson 95% intervals describe binomial proportions conditional on the sampled source records and do not account for ascertainment, upstream predictor dependence, or transport to unresolved variants.

The ≥3-of-4 endpoint supplies conventional sensitivity, specificity, PPV, NPV, F1, MCC, and balanced accuracy among evaluable records. Denominators and incomplete counts are retained by source label and gene. V0/V4 agreement is reported jointly with unanimity coverage. Opposite-direction proportions use all source anchors within each label. Comparator AUROCs are calculated on matched evaluable records. Stable rescue requires correct ensemble direction and incorrect comparator direction in all five outer-test appearances, with comparator cut-points fitted separately in each outer-training partition. For each source-label group, the rescue denominator includes all anchors with an observed native comparator score, including incomplete records. This measure reports rescue yield in the native-score-available source cohort.

Analyses used Python 3.12.13, NumPy 2.3.5, pandas 2.2.3, scikit-learn 1.8.0, SciPy 1.17.0, and joblib 1.5.3; scientific figures were generated with Matplotlib 3.10.8. The separately archived local catalogue-viewer runtime used R 4.4.3, shiny 1.14.0, DT 0.34.0, jsonlite 2.0.0, and rsconnect 1.11.0. Split seeds start at 20,260,723 and modelling seeds at 20260714, with deterministic offsets recorded in the scripts. The reproducibility package includes environment versions, fold maps, input hashes, feature-selection audits, and 58 fitted model objects. The archived replay audit regenerated assessment outputs and all 43,679 application outputs from these objects, with a maximum absolute difference of 1.11 × 10^−16^.

## Figures and Tables

**Table 1:** Deployment panel and selection frequency across 25 outer-training fits.

Rank	Predictor	Category	Outer Folds	Frequency
1	MetaRNN_rankscore	Variant-effect score	24	96%
2	am_pathogenicity	Variant-effect score	25	100%
3	CADD_PHRED	Variant-effect score	25	100%
4	PHACTboost_rankscore	Variant-effect score	25	100%
5	gMVP_rankscore	Variant-effect score	22	88%
6	MutationTaster_rankscore	Variant-effect score	25	100%
7	REVEL_rankscore	Variant-effect score	20	80%
8	Eigen-raw_coding_rankscore	Variant-effect score	25	100%
9	VARITY_R_LOO_rankscore	Variant-effect score	18	72%
10	ESM1b_converted_rankscore	Variant-effect score	25	100%
11	PrimateAI_rankscore	Variant-effect score	25	100%
12	fathmm-XF_coding_rankscore	Variant-effect score	25	100%
13	MVP_rankscore	Variant-effect score	25	100%
14	MutationAssessor_rankscore	Variant-effect score	25	100%
15	GERP++_RS_rankscore	Conservation/context	25	100%
16	phyloP100way_vertebrate_rankscore	Conservation/context	25	100%
17	phyloP470way_mammalian_rankscore	Conservation/context	25	100%
18	phastCons100way_vertebrate_rankscore	Conservation/context	25	100%
19	SpliceAI_DS_max	Splicing	25	100%
20	EVE_SCORE	Variant-effect score	25	100%
21	MetaSVM_rankscore	Variant-effect score	25	100%
22	DEOGEN2_rankscore	Variant-effect score	24	96%
23	MetaLR_rankscore	Variant-effect score	9	36%
24	bStatistic_converted_rankscore	Conservation/context	10	40%
25	MutFormer_rankscore	Variant-effect score	22	88%

**Table 2:** Fitted deployment hyperparameters, calibration coefficients, and vote thresholds.

Component	Role	Hyperparameters	Intercept	Slope	Threshold	Interpretation
SVM–RBF	Base learner	C = 6; gamma = scale	0.04250	2.71178	0.5000	One vote
L2 logistic	Base learner	C = 1; L2 penalty	−0.01277	0.93765	0.6897	One vote
Extra Trees	Base learner	220 trees; depth 18; max features 0.7; min leaf 1	0.01758	1.22101	0.5905	One vote
HistGB	Base learner	31 leaves; min leaf 20; 100 iterations; rate 0.1; L2 = 0	−0.02603	0.53038	0.5000	One vote
Mean4	Mean score	Four calibrated probabilities	—	—	—	No vote
S_MAC_	Meta-calibration	Logit(Mean4)	0.03388	1.13536	—	Ranking only

**Table 3:** First-repeat out-of-fold vote groups and incomplete-record accounting.

Source Label *	V0	V1–V3	V4	Incomplete	Total
B/LB	1046	45	16	80	1187
LP/P	32	33	874	34	973
Total	1078	78	890	114	2160

* V0 and V4 denote unanimous B/LB- and LP/P-directed votes, respectively; V1–V3 denotes mixed votes. Incomplete records failed the annotation-completeness rule. Unanimity coverage uses all 2160 source anchors; conditional source-direction agreement uses the 1968 unanimous outputs.

**Table 4:** Internal sensitivity analyses with explicit total and evaluable denominators.

Analysis	Total n	Scored n	BA	AUROC	AUPRC	Evaluation
Primary, five repeats	2160	2046	0.9604 ± 0.0023	0.9909 ± 0.0013	0.9912 ± 0.0013	Mean ± split SD
Primary, repeat 1	2160	2046	0.9592	0.9886	0.9890	First-repeat folds
With Reliability_index	2160	2046	0.9614	0.9891	0.9895	First-repeat folds
Candidate-pool exclusion of addAF + AlphaMissense	2160	2046	0.9607	0.9896	0.9904	First-repeat folds
Current-missense refit	2120	2019	0.9615	0.9902	0.9906	First-repeat folds
Primary, same missense subset	2120	2019	0.9584	0.9884	0.9887	First-repeat folds
Leave one gene out	2160	2045	0.9453	0.9812	0.9810	17 complete gene holdouts

**Table 5:** Complete hospital-list audit of 31 distinct missense variants within the 17-gene scope.

Gene	MANE Description	Source Rows	Observed/25	Record Origin	Votes	V	S_MAC_
*BRCA1*	c.53T>C; p.Met18Thr	1	25	Anchor	1111	V4	0.9922
*BRCA1*	c.181T>G; p.Cys61Gly	1	25	Anchor	1111	V4	0.9977
*BRCA1*	c.5072C>G; p.Thr1691Arg	1	24	Anchor	1111	V4	0.9808
*BRCA1*	c.5074G>C; p.Asp1692His	1	24	Anchor	1111	V4	0.9982
*BRCA1*	c.5096G>A; p.Arg1699Gln	1	25	Anchor	1111	V4	0.9906
*BRCA1*	c.5123C>A; p.Ala1708Glu	2	25	Anchor	1111	V4	0.9976
*BRCA1*	c.5497G>A; p.Val1833Met	1	25	Anchor	1111	V4	0.9861
*BRCA2*	c.8177A>G; p.Tyr2726Cys	1	22	Anchor	1111	V4	0.9789
*BRCA2*	c.8351G>A; p.Arg2784Gln	1	22	Catalogue	1101	V3	0.7156
*BRCA2*	c.8524C>T; p.Arg2842Cys	1	22	Catalogue	1111	V4	0.9345
*BRCA2*	c.9371A>T; p.Asn3124Ile	2	22	Anchor	1111	V4	0.9815
*PALB2*	c.3113G>C; p.Trp1038Ser	1	23	Additional	1111	V4	0.9695
*ATM*	c.875C>T; p.Pro292Leu	1	25	Anchor	1111	V4	0.9780
*ATM*	c.967A>G; p.Ile323Val	1	25	Anchor	1111	V4	0.9947
*ATM*	c.6047A>G; p.Asp2016Gly	3	25	Anchor	1111	V4	0.9978
*ATM*	c.6154G>A; p.Glu2052Lys	2	25	Catalogue	0001	V1	0.2646
*ATM*	c.6188G>A; p.Gly2063Glu	2	25	Catalogue	1111	V4	0.9867
*ATM*	c.6679C>T; p.Arg2227Cys	1	25	Anchor	1111	V4	0.9937
*ATM*	c.8122G>A; p.Asp2708Asn	1	25	Anchor	1111	V4	0.9632
*CHEK2*	c.190G>A; p.Glu64Lys	2	24	Catalogue	0000	V0	0.0947
*CHEK2*	c.427C>T; p.His143Tyr	1	24	Catalogue	1111	V4	0.9858
*CHEK2*	c.433C>T; p.Arg145Trp	3	24	Anchor	1111	V4	0.9883
*CHEK2*	c.470T>C; p.Ile157Thr	5	24	Catalogue	0000	V0	0.0093
*CHEK2*	c.499G>A; p.Gly167Arg	3	24	Anchor	1111	V4	0.9909
*CHEK2*	c.557A>G; p.Asn186Ser	1	24	Catalogue	0000	V0	0.0040
*TP53*	c.473G>A; p.Arg158His	1	25	Anchor	1111	V4	0.9930
*TP53*	c.742C>T; p.Arg248Trp	1	25	Anchor	1111	V4	0.9988
*TP53*	c.818G>A; p.Arg273His	1	25	Anchor	1111	V4	0.9901
*TP53*	c.856G>A; p.Glu286Lys	1	25	Anchor	1111	V4	0.9948
*RAD51C*	c.414G>C; p.Leu138Phe	2	25	Anchor	1011	V3	0.8903
*FANCA*	c.4009A>G; p.Ser1337Gly	1	24	Anchor	1111	V4	0.9597

All 47 in-scope source rows are represented. Votes are ordered SVM–RBF, logistic regression, Extra Trees, and HistGB. Anchor: variant used to fit the deployment model (22; displayed scores are in-sample). Catalogue: existing application record (8). Additional: variant outside the fitted anchor and application sets, with a supplied numeric annotation record (1). All 31 variants pass ≥20/25 completeness. MANE Select descriptions are shown; RefSeq versions, genomic identities and original source strings are retained in the accompanying row-level audit. Source-row counts are not patient counts.

**Table 6:** Prespecified 17-gene source counts.

Gene	B/LB	LP/P
*ATM*	67	75
*BARD1*	24	3
*BLM*	21	6
*BRCA1*	347	217
*BRCA2*	306	84
*BRIP1*	25	7
*CDH1*	89	20
*CHEK2*	8	9
*FANCA*	63	77
*FANCC*	15	4
*NBN*	18	2
*PALB2*	34	6
*PTEN*	22	211
*RAD51C*	10	9
*RAD51D*	10	1
*STK11*	7	33
*TP53*	121	209
Total	1187	973

**Table 7:** Hyperparameter grids for the four fixed learner families.

Learner	Search Space
L2 logistic regression	C: 0.03, 0.1, 0.3, 1, 3, 10; l1_ratio = 0
SVM–RBF	C: 0.25, 0.75, 2, 6, 16; gamma: scale, 0.01, 0.03, 0.1
Extra Trees	220 trees; max depth: none, 10, 18; max features: sqrt, 0.4, 0.7; min leaf: 1, 3, 7
HistGB	Max leaves: 15, 31; min leaf: 10, 20; iterations: 100, 200; learning rate: 0.03, 0.1; L2: 0, 1

Note. Six unique configurations per learner and training partition were sampled without replacement. Extra Trees used 220 trees. Hyperparameters were ranked by mean inner AUROC, its sample SD, and a deterministic parameter-string tie-break.

## Data Availability

The data presented in this study are openly available in HBOCpred at https://alaymd.shinyapps.io/hbocpred/ (accessed on 15 September 2026).

## Supplementary Materials

The following supporting information can be downloaded at: https://www.mdpi.com/article/10.3390/ijms27198865/s1.

## Abbreviations

ACMG/AMP, American College of Medical Genetics and Genomics/Association for Molecular Pathology; AUPRC, area under the precision–recall curve; AUROC, area under the receiver operating characteristic curve; B/LB, benign/likely benign; HistGB, histogram-based gradient boosting; LOGO, leave-one-gene-out; LP/P, likely pathogenic/pathogenic; MAC, modified adaptive classifier; MANE, Matched Annotation from NCBI and EMBL-EBI; MCC, Matthews correlation coefficient; NPV, negative predictive value; NR, review-status annotation not recorded; OOF, out-of-fold; PPV, positive predictive value; S_MAC_, continuous modified adaptive classifier score; SMD, standardised mean difference; SVM–RBF, support vector machine with radial basis function kernel; V0–V4, audit states defined by 0–4 LP/P-directed votes; VUS, variant of uncertain significance.
