# Supplementary material for: HBOCpred: An Ensemble Framework for Prioritisation of Germline Missense Variants in Hereditary Breast and Ovarian Cancer
Source: Int J Mol Sci. 2026 Oct 5;27(19):8865. doi: 10.3390/ijms27198865 (PMC13665518; doi:10.3390/ijms27198865)
Supplement: Supplementary file 1 [file ijms-27-08865-s001.zip › HBOCpred_Supplementary.pdf]

Supplementary Materials

HBOCpred: an ensemble framework for prioritisation of germline missense variants in hereditary breast and ovarian cancer.

These materials report the per-gene results, conditional intervals and source-record audits accompanying HBOCpred. The development cohort contains 2,160 source-labelled anchors; the application catalogue contains 43,679 unresolved records. Tables S1–S5 and Figure S1 are supported by Supplementary Data S1 (HBOCpred_Reproducibility.zip). The requested 25-predictor anchor matrix is also available separately.

S1 Evaluability across repeated cross validation

Completeness was recalculated from the original numeric matrix using each outer fitted model's selected panel and the threshold ceil(0.8 × panel size). All 10,800 stored record-level flags matched the recalculation. The same 2,046 anchors were evaluable in all five repeats and the same 114 were incomplete; no variant changed evaluability. All 43,679 application records met the deployment completeness rule.

Table S1. Class-specific evaluability, identical in each of the five repeats.

Analysis subset	Status	B/LB	LP/P	Total
All anchors	Source	1,187	973	2,160
All anchors	Evaluable	1,107	939	2,046
All anchors	Incomplete	80	34	114
ClinVar-concordant	Source	1,083	915	1,998
ClinVar-concordant	Evaluable	1,081	904	1,985
ClinVar-concordant	Incomplete	2	11	13

The ClinVar-concordant subset was selected solely by agreement of embedded clinical significance with the processed source label. Its evaluable identities were also identical across repeats. Mean balanced accuracy was 0.9598 ± 0.0022 and AUROC was 0.9907 ± 0.0013 across the five repeats. These SDs describe split stability.

S2 Gene held out and within gene evaluation

Each gene was excluded from every fitting stage of its corresponding model. Table S2A reports the secondary ≥3-of-4 endpoint and AUROC within each held-out gene. Table S2B reports primary repeated-cross-validation AUROCs calculated separately within each gene. The tables use different fitted models and retain their respective evaluable denominators.

Table S2A. Leave-one-gene-out evaluation with source and evaluable counts, class-specific results and gene-held-out AUROC.

Gene	Source n	Scored n	LP/P correct / scored	Sensitivity (95% interval)	B/LB correct / scored	Specificity (95% interval)	Gene-held-out AUROC
BRCA1	564	528	193/207	93.2% (89.0–95.9)	306/321	95.3% (92.4–97.1)	0.9842
BRCA2	390	383	77/81	95.1% (88.0–98.1)	289/302	95.7% (92.8–97.5)	0.9900
PALB2	40	39	5/6	83.3% (43.6–97.0)	33/33	100.0% (89.6–100.0)	0.9848
ATM	142	134	63/69	91.3% (82.3–96.0)	62/65	95.4% (87.3–98.4)	0.9924
CHEK2	17	17	7/9	77.8% (45.3–93.7)	8/8	100.0% (67.6–100.0)	0.9722
TP53	330	313	192/203	94.6% (90.6–96.9)	109/110	99.1% (95.0–99.8)	0.9899
PTEN	233	212	207/207	100.0% (98.2–100.0)	5/5	100.0% (56.6–100.0)	1.0000
CDH1	109	109	18/20	90.0% (69.9–97.2)	80/89	89.9% (81.9–94.6)	0.9691
STK11	40	39	32/32	100.0% (89.3–100.0)	7/7	100.0% (64.6–100.0)	1.0000
BARD1	27	25	2/2	100.0% (34.2–100.0)	23/23	100.0% (85.7–100.0)	1.0000
BRIP1	32	32	7/7	100.0% (64.6–100.0)	25/25	100.0% (86.7–100.0)	1.0000
RAD51C	19	18	7/8	87.5% (52.9–97.8)	10/10	100.0% (72.2–100.0)	0.9750
RAD51D	11	11	1/1	100.0% (20.7–100.0)	10/10	100.0% (72.2–100.0)	1.0000
NBN	20	19	0/2	0.0% (0.0–65.8)	17/17	100.0% (81.6–100.0)	0.8235
BLM	27	27	6/6	100.0% (61.0–100.0)	21/21	100.0% (84.5–100.0)	1.0000
FANCA	140	121	50/75	66.7% (55.4–76.3)	46/46	100.0% (92.3–100.0)	0.9907
FANCC	19	18	4/4	100.0% (51.0–100.0)	14/14	100.0% (78.5–100.0)	1.0000

Sensitivity and specificity intervals are Wilson 95% intervals conditional on the evaluated records. AUROC uses the continuous S_MAC score within each held-out gene. Incomplete counts equal source n minus scored n. PTEN specificity is based on five evaluable B/LB anchors; 17 further B/LB anchors are incomplete.

In the primary repeated cross-validation analysis, AUROC restricted to LP/P–B/LB pairs from the same gene was 0.9877 ± 0.0016 across five repeats (125,091 pairs per repeat). The unweighted mean of the 17 gene AUROCs was 0.9734 ± 0.0103. These SDs describe split stability, and the analysis does not remove within-gene ascertainment or predictor–label dependence. Table S2B displays the existing gene-level results

from tables/within_gene_oof_summary.csv; aggregate summaries are retained in tables/within_gene_aggregate_summary.csv.

Table S2B. Primary out-of-fold discrimination within each gene across five repeats.

Gene	B/LB evaluable n	LP/P evaluable n	Within-gene AUROC Mean ± split SD
BRCA1	321	207	0.9862 ± 0.0019
BRCA2	303	81	0.9886 ± 0.0035
PALB2	33	6	0.9899 ± 0.0062
ATM	65	69	0.9908 ± 0.0010
CHEK2	8	9	0.9611 ± 0.0152
TP53	110	203	0.9905 ± 0.0007
PTEN	5	207	1.0000 ± 0.0000
CDH1	89	20	0.9756 ± 0.0054
STK11	7	32	1.0000 ± 0.0000
BARD1	23	2	1.0000 ± 0.0000
BRIP1	25	7	1.0000 ± 0.0000
RAD51C	10	8	0.9825 ± 0.0112
RAD51D	10	1	1.0000 ± 0.0000
NBN	17	2	0.6882 ± 0.1772
BLM	21	6	1.0000 ± 0.0000
FANCA	46	75	0.9935 ± 0.0020
FANCC	14	4	1.0000 ± 0.0000

Counts refer to the primary evaluable cohort and are identical across the five repeats. The SD measures split stability, not a confidence interval. These results are distinct from the gene-held-out estimates in Table S2A; no model was refitted to create this table.

S3 gnomAD candidate eligibility

The 50 gnomAD candidates were matched by complete GRCh38 genomic allele against the development and application cohorts. Forty-five matched anchors, one matched the application catalogue and four matched neither cohort. Multinucleotide alleles were retained as distinct variants. We reviewed cached same-gene transcript consequences and available original numeric annotations for the four remaining candidates. Only the TP53 candidate met the deployment completeness rule and was scored with the unchanged frozen model (Table S3). No benign-set performance estimate was calculated.

Table S3. Four gnomAD candidates absent from both study cohorts.

Gene	GRCh38 allele	Transcript consequence	Inputs and output	Source labels
TP53	17-7676154-G-C	MANE NM_000546.6: c.215C>G; p.Pro72Arg	24/25; V0 (0000) S_MAC 0.0007	Processed VUS; embedded ClinVar Benign (2-star)
BRCA2	13-32398747-A-G	MANE NM_000059.4: c.10234A>G; p.Ile3412Val	1/25; not scored	Original annotation row: N4BP2L1, LB; embedded ClinVar absent
CHEK2	22-28712124-A-C	Alternative ENST00000464581.7: c.11T>G; p.Leu4Arg. MANE: intronic	Matched inputs unavailable; not scored	No matched source row
PTEN	10-87864144-G-C	Alternative ENST00000693560.1: c.194G>C; p.Cys65Ser. MANE: 5′ UTR	Matched inputs unavailable; not scored	No matched source row

The *TP53* allele was absent from the training identity lists of all 58 fitted objects. Its source VUS label and embedded ClinVar Benign assertion are retained separately; no reference label was changed. The candidate was not added to either study cohort, whose denominators remain 2,160 and 43,679. The *BRCA2* allele is MANE missense in the current snapshot, but its original annotation row supplies only one of the 25 deployment predictors. Full frequency, transcript, cohort and scoring records are provided in audit/cohort_verification/. An alternate-allele-frequency screen alone does not complete BA1 adjudication. Frequency-based selection may share information with model inputs, and absence from the study cohorts does not establish independence from clinical labels or constituent-predictor training data.

Methodological source: Ghosh et al. Updated recommendation for the benign stand-alone ACMG/AMP criterion. Hum. Mutat. 2018;39:1525–1530. doi:10.1002/humu.23642.

S4 Conditional intervals for the first repeat

We used a class-stratified bootstrap with one fixed out-of-fold prediction per evaluable variant, 2,000 resamples and seed 20260905. Sensitivity, specificity and balanced accuracy use the secondary ≥3-of-4 endpoint; AUROC, AUPRC and Brier score use S_MAC. These intervals condition on the fitted scores and the sampled source-label classes. They exclude model refitting, gene clustering and historical model-selection uncertainty, and do not account for source-label circularity or transport to unresolved variants. The five-repeat SDs reported in the manuscript describe split stability.

Table S4. Conditional first-repeat intervals on 2,046 evaluable anchors.

Metric	First-repeat estimate	Conditional 2.5th–97.5th percentiles
Sensitivity	0.9446	0.9297–0.9585
Specificity	0.9738	0.9639–0.9828
Balanced accuracy	0.9592	0.9504–0.9675

Metric	First-repeat estimate	Conditional 2.5th–97.5th percentiles
AUROC	0.9886	0.9838–0.9928
AUPRC	0.9890	0.9849–0.9929
Brier score	0.0307	0.0253–0.0366

S5 TRIPOD AI reporting map

Table S5 maps the study to the TRIPOD+AI reporting items and identifies remaining gaps; it does not claim complete compliance. The analysis unit is a variant.

Table S5. Study-specific TRIPOD+AI reporting map.

Item	Topic	Location	Coverage / limitation
1–2	Title and abstract	Title; Abstract	Model, target, outputs, evaluability and internal scope stated.
3a–c	Context and users	Introduction; Discussion	Research-curator use specified; ancestry/demographic representativeness unavailable.
4	Objectives	Introduction final paragraph; 4.1	Development and internal evaluation; clinical validation withdrawn.
5a–b	Source data and dates	4.1–4.3; audit/input_manifest.json; audit/cache	Source archives hashed; 2026-09-05 audit snapshots retained. Historical source release dates unavailable.
6a–c	Eligibility and setting	4.1–4.2; Table 6	Variant-level scope; historical transcript discrepancies declared; treatment/follow-up not applicable.
7	Preparation	4.3; prepare_inputs.py	Numeric parsing, semantic reduction and partition-specific preprocessing documented.
8a–c	Reference labels	4.2; Discussion; source metadata	Processed y distinguished from embedded ClinVar labels. Independent blinded outcome adjudication unavailable.
9a–c	Predictors	4.3; Table 1; documentation/data_dictionary.md	47 primary candidates and 25 deployment predictors supplied. Upstream training provenance remains incomplete.
10	Sample size	4.1; Tables 3, 4 and 6	All available source anchors used; no formal prospective calculation; small gene strata acknowledged.
11	Missingness	2.2; 4.2–4.3; output flags	Median imputation and identical completeness suppression; all denominators retained.
12a–g	Analysis design	4.4–4.9; scripts; fold maps	Nested fitting, tuning, calibration, thresholding, metrics and sensitivity analyses explicit.
13	Class balance	4.1; fitted learner settings	No class resampling or class-weight adjustment; class counts and calibration reported.
14	Fairness	3.1 Limitations; data dictionary	Ancestry-specific and demographic fairness not estimable from these source data.
15	Outputs	4.2 and 4.6; Table 3	Shared vote descriptors; retrospective convention; S_MAC ranking; completeness guard.
16	Population differences	2.7; 4.8; Figure 6	Predictor shift, disagreement shift, gene information and absent application outcomes documented.
17	Ethics	Ethics and consent statements	Source hospital approval retained; no new external clinical outcome analysis.
18a–b	Funding and interests	Funding; Author Contributions; conflicts statement	No external funding declared. Author contributions and conflicts are stated in the manuscript.

Item	Topic	Location	Coverage / limitation
18c–d	Protocol and registration	4.1; documentation/executed_protocol.md	Revision-stage reanalysis not prospectively registered; executed protocol and deviations supplied.
18e–f	Data and code access	Availability statements; Supplementary Data S1	The full archive and standalone matrix have direct access links. The code repository is public. A DOI-bearing deposition has not yet been established.
19	Public involvement	Reporting information unavailable	Patient/public involvement is not documented in the study records.
20a–c	Cohort accounting	Tables 3 and 6; Figures 2, 6 and 8	Source and evaluable counts, application population and missingness distinguished; clinical demographics unavailable.
21	Analysis denominators	Tables 3 and 4; Tables S1 and S2; fit audits	Training and held-out identities, class denominators and incomplete flags are provided. Evaluable variant identities were identical across all five repeats.
22	Complete model	Table 2; models/deployment.joblib; Supplementary Data S1	Preprocessors, parameters, calibrators, thresholds, fitted models and scoring code are supplied in the linked archive.
23a–b	Performance and variation	2.2–2.7; Figures 1–6; Tables S2 and S4	Per-gene AUROC and Wilson intervals, repeat SDs and conditional intervals for fixed scores are reported. Prospective and transport intervals are unavailable.
24	Model revision	2.1; README; documentation/executed_protocol.md	New fits supersede prior numerical estimates; old missing fits are not reconstructed or claimed verified.
25–26	Interpretation and limits	3 Discussion; 3.1 Limitations; 3.2 Conclusions	Source-label dependence, gene confounding, distribution shift, cost asymmetry, missingness and transcript limits are discussed.
27a–c	Use and next evaluation	3 Discussion; 3.2 Conclusions; 4.2; viewer notes	Expert research use; build and transcript checks; incomplete-output suppression. Independent evidence and clinical utility evaluation remain necessary.

Checklist: Collins et al. TRIPOD+AI. BMJ 2024;385:e078378. doi:10.1136/bmj-2023-078378. Expanded checklist: https://www.tripod-statement.org/wp-content/uploads/2024/04/TRIPODAI-Supplement.pdf.

S6 Source provenance

Current Ensembl annotation identifies 2,039 MANE missense anchors, 81 additional same-gene alternative-transcript missense anchors and 40 without a current same-gene missense consequence, all within the original 2,160 anchors. Of these 40, 13 failed the primary completeness rule and 27 remained evaluable in every repeat. The sensitivity refit on 2,120 current missense anchors is reported separately. The hospital source list contains 122 rows. Of these, 75 fall outside the 17-gene scope; the 47 in-scope rows resolve to 31 distinct missense variants. Twenty-two overlap anchors, eight overlap the application catalogue and one additional variant is absent from both cohorts. All 31 meet the ≥20/25 deployment completeness rule. These are source-row and variant counts, not patient counts. Repeated records of the same genomic allele were collapsed for variant-level scoring. Anchor-overlapping deployment scores are in-sample, and clinical reference labels were not independently adjudicated. Transcript identity was checked against Ensembl snapshots and NCBI ClinVar VCV000005591.136 (https://www.ncbi.nlm.nih.gov/clinvar/variation/5591/; accessed 6 September 2026). CHEK2 Ile200Thr/Ile157Thr and Gly210Arg/Gly167Arg were each merged by genomic allele. A bare c.599T>C catalogue lookup would select p.Val200Ala on MANE Select. The full row audit preserves source names, transcript strings and discrepant ATM rsIDs. Additional Ensembl responses are in audit/cache/hospital_additional_transcripts.json.gz. These checks establish allele identity, not clinical reference classifications.

Supplementary Data S1 Reproducibility archive

HBOCpred_Reproducibility.zip contains the processed matrices, fitted models and scripts needed to inspect and replay the reported analyses.

- **data/anchors_all_numeric.csv** — 2,160 anchors, with 90 numeric fields and nine metadata columns.
- **data/anchor_deployment_panel.csv** — The 25 deployment predictors for all 2,160 anchors, with SPDI, Gene and y (0 = B/LB; 1 = LP/P). Empty predictor cells retain missing annotations before training-specific imputation; they are not zeroes.
- **outputs/fold_assignments.csv** — 10,800 record assignments across the five outer-test repeats.
- **outputs/oof_predictions_repeated_cv.csv** — 10,800 repeated out-of-fold records, including incomplete-output flags.
- **outputs/logo_predictions.csv** — Leave-one-gene-out outputs for all 2,160 anchors.
- **data/application_deployment_panel.csv** — The 25-predictor matrix for the 43,679 unresolved records, with identifiers.
- **outputs/application_catalogue.csv** — The complete deployment catalogue for all 43,679 unresolved records.
- **documentation/data_dictionary.md** — Column definitions and interpretation of the released records.
- **documentation/CSV_INDEX.csv** — An index of the CSV files provided in the archive.

The archive contains 58 fitted objects: 25 cross-validation models, 17 gene-held-out models, 15 sensitivity-analysis models and one deployment model. Each fit audit records training identities and inner fold memberships. Assessment outputs retain learner probabilities and votes, V-state, Mean4, S_MAC and completeness alongside source identifiers. Replaying the saved models reproduced the assessment and application probabilities to a maximum absolute difference of 1.11×10^{-16}. The cohort audit also recalculated completeness for all 10,800 repeated out-of-fold records and replayed the unchanged deployment model before scoring the additional TP53 candidate.

Code and the processed anchor matrix are accessible in the public HBOCpred repository. All 58 fitted objects are provided in the full archive linked above.
