# Supplementary material for: HBOCpred: An Ensemble Framework for Prioritisation of Germline Missense Variants in Hereditary Breast and Ovarian Cancer
Source: Int J Mol Sci. 2026 Oct 5;27(19):8865. doi: 10.3390/ijms27198865 (PMC13665518; doi:10.3390/ijms27198865)
Supplement: Supplementary file 1 [file ijms-27-08865-s001.zip › HBOCpred_Reproducibility.zip!/HBOCpred/figures/Figure1_workflow.pdf]

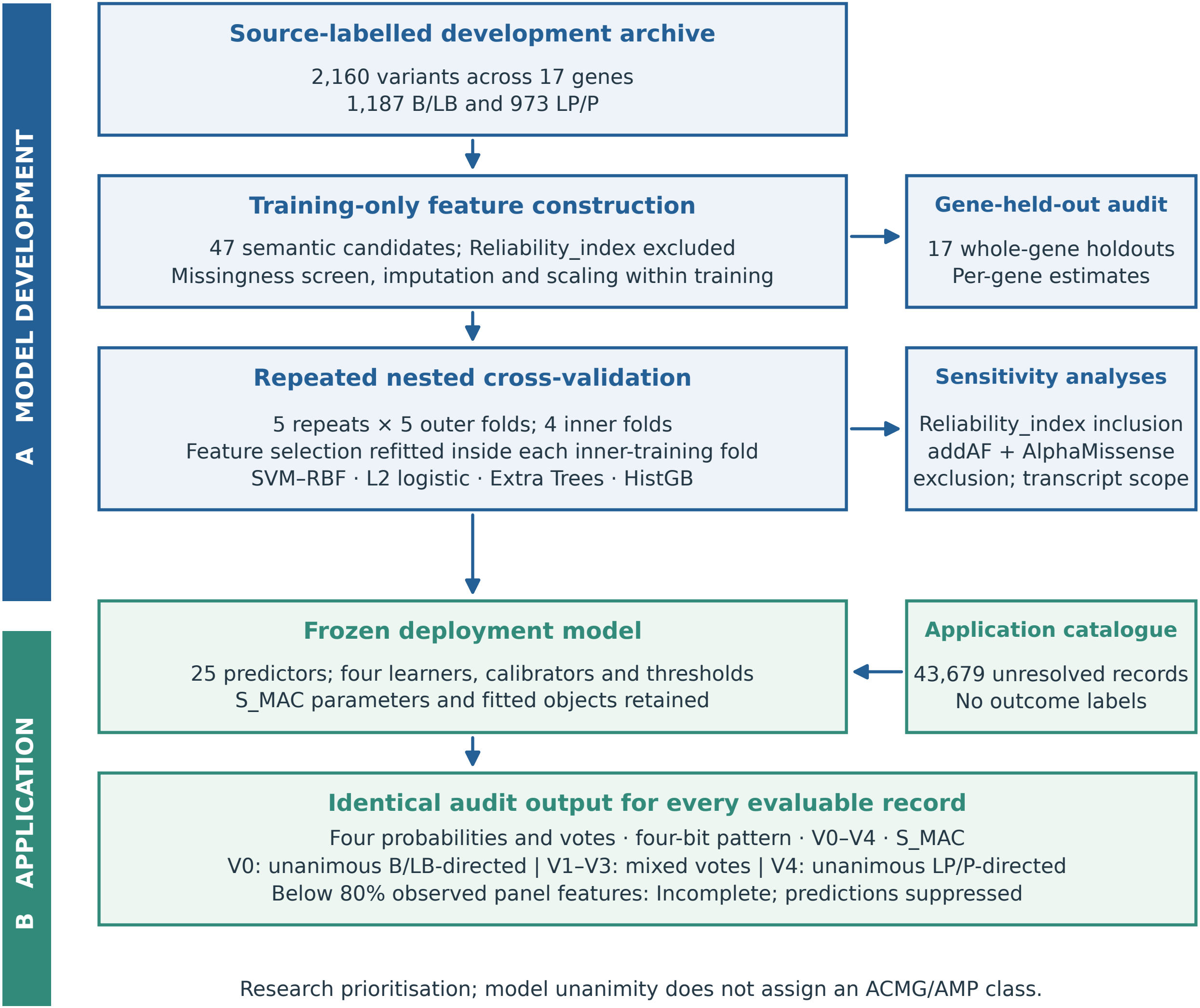
A MODEL DEVELOPMENT
Source-labelled development archive
2,160 variants across 17 genes
1,187 B/LB and 973 LP/P
Training-only feature construction
47 semantic candidates; Reliability_index excluded
Missingness screen, imputation and scaling within training
Gene-held-out audit
17 whole-gene holdouts
Per-gene estimates
Repeated nested cross-validation
5 repeats × 5 outer folds; 4 inner folds
Feature selection refitted inside each inner-training fold
SVM–RBF · L2 logistic · Extra Trees · HistGB
Sensitivity analyses
Reliability_index inclusion
addAF + AlphaMissense
exclusion; transcript scope
B APPLICATION
Frozen deployment model
25 predictors; four learners, calibrators and thresholds
S_MAC parameters and fitted objects retained
Application catalogue
43,679 unresolved records
No outcome labels
Identical audit output for every evaluable record
Four probabilities and votes · four-bit pattern · V0–V4 · S_MAC
V0: unanimous B/LB-directed | V1–V3: mixed votes | V4: unanimous LP/P-directed
Below 80% observed panel features: Incomplete; predictions suppressed
Research prioritisation; model unanimity does not assign an ACMG/AMP class.
