# Supplementary material for: HBOCpred: An Ensemble Framework for Prioritisation of Germline Missense Variants in Hereditary Breast and Ovarian Cancer
Source: Int J Mol Sci. 2026 Oct 5;27(19):8865. doi: 10.3390/ijms27198865 (PMC13665518; doi:10.3390/ijms27198865)
Supplement: Supplementary file 1 [file ijms-27-08865-s001.zip › HBOCpred_Reproducibility.zip!/HBOCpred/figures/Figure2_performance.pdf]

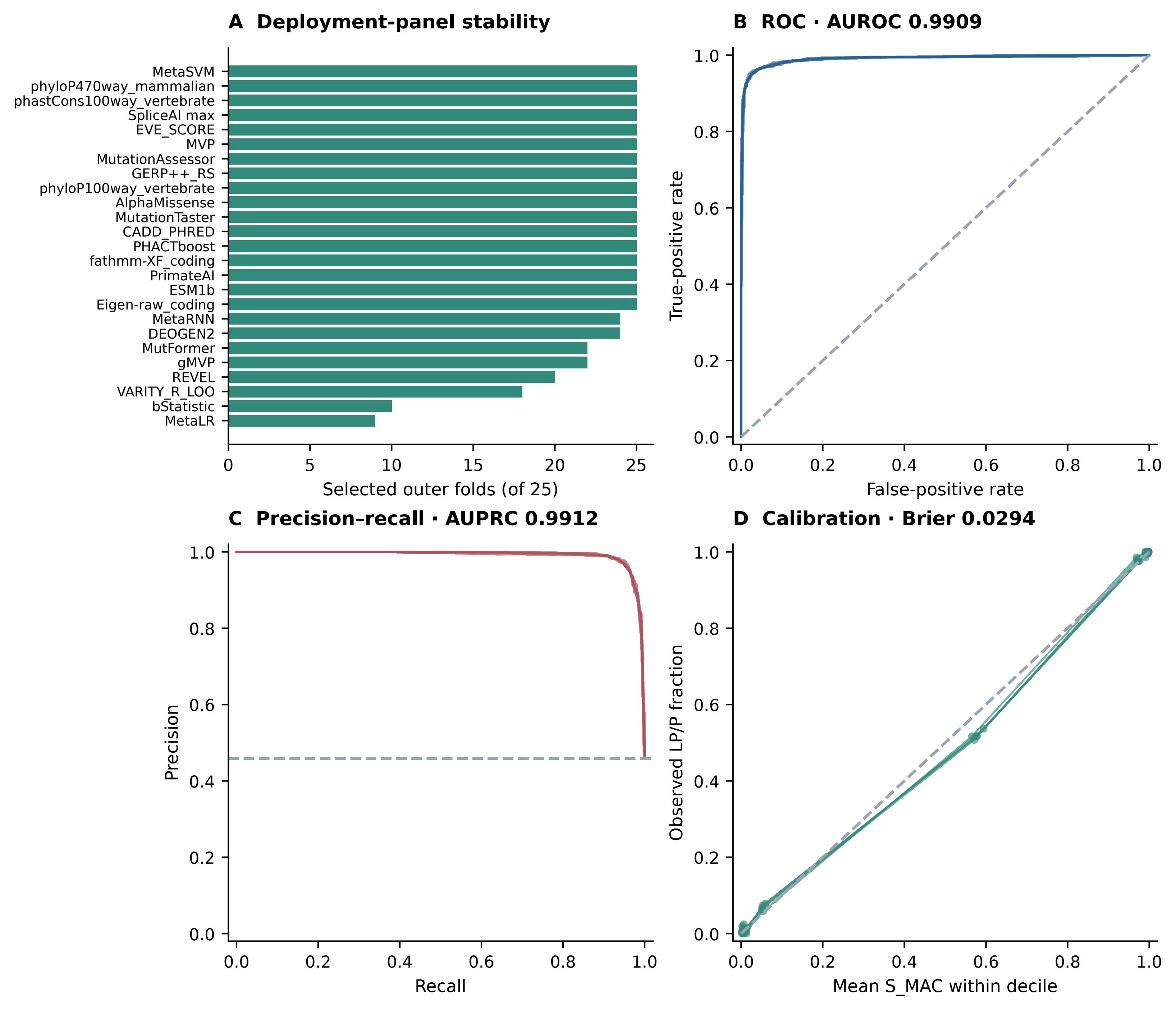
A Deployment-panel stability
MetaSVM
phyloP470way_mammalian
phastCons100way_vertebrate
SpliceAI max
EVE_SCORE
MVP
MutationAssessor
GERP++_RS
phyloP100way_vertebrate
AlphaMissense
MutationTaster
CADD_PHRED
PHACTboost
fathmm-XF_coding
PrimateAI
ESM1b
Eigen-raw_coding
MetaRNN
DEOGEN2
MutFormer
gMVP
REVEL
VARITY_R_LOO
bStatistic
MetaLR
0 5 10 15 20 25
Selected outer folds (of 25)
B ROC · AUROC 0.9909
True-positive rate
False-positive rate
0.0 0.2 0.4 0.6 0.8 1.0
C Precision–recall · AUPRC 0.9912
Precision
Recall
0.0 0.2 0.4 0.6 0.8 1.0
D Calibration · Brier 0.0294
Observed LP/P fraction
Mean S_MAC within decile
0.0 0.2 0.4 0.6 0.8 1.0
