# Supplementary material for: HBOCpred: An Ensemble Framework for Prioritisation of Germline Missense Variants in Hereditary Breast and Ovarian Cancer
Source: Int J Mol Sci. 2026 Oct 5;27(19):8865. doi: 10.3390/ijms27198865 (PMC13665518; doi:10.3390/ijms27198865)
Supplement: Supplementary file 1 [file ijms-27-08865-s001.zip › HBOCpred_Reproducibility.zip!/HBOCpred/figures/Figure3_vstates.pdf]

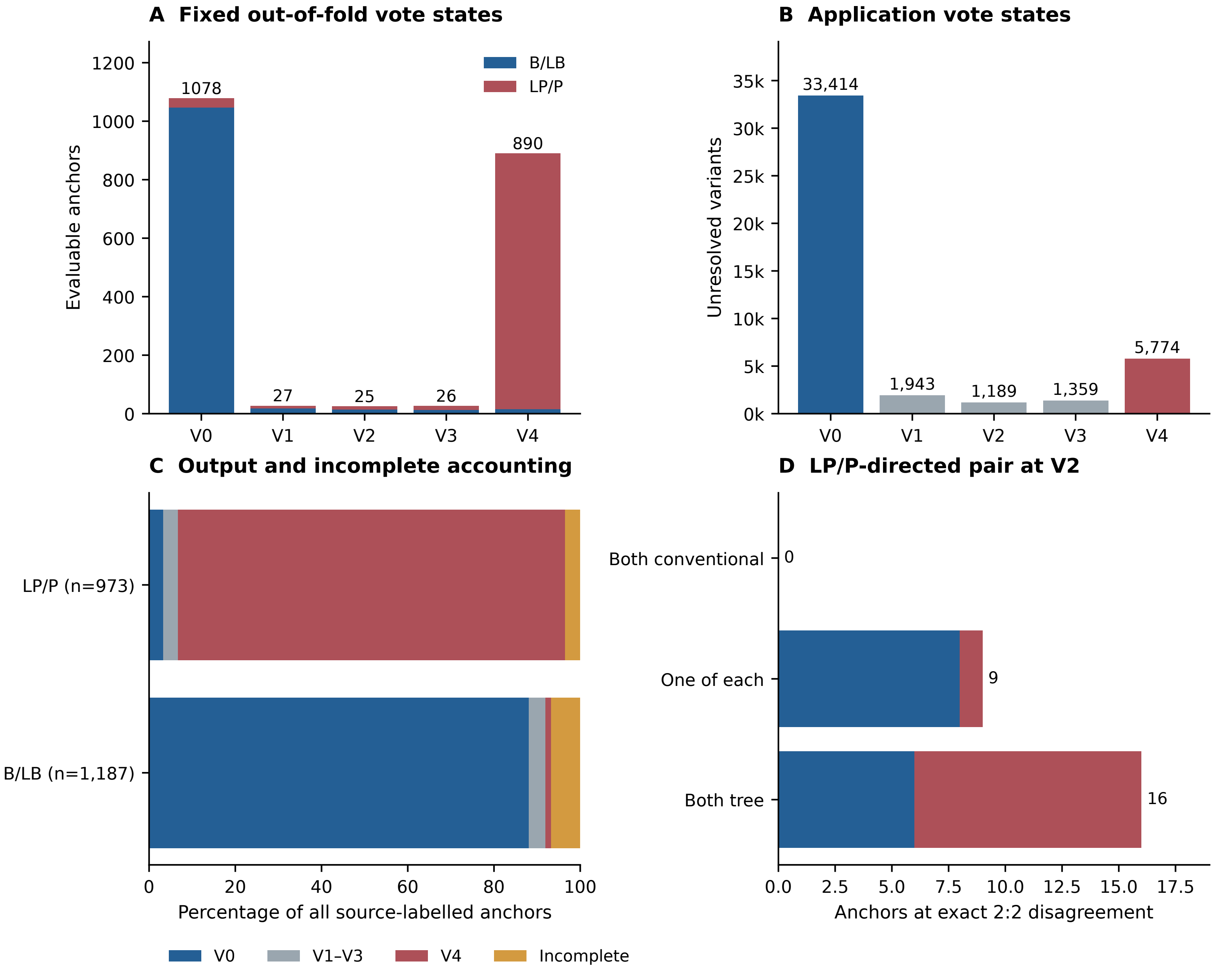

A Fixed out-of-fold vote states
B/LB
LP/P
Evaluable anchors
0
200
400
600
800
1000
1200
1078
27
25
26
890
V0
V1
V2
V3
V4
B Application vote states
Unresolved variants
0k
5k
10k
15k
20k
25k
30k
35k
33,414
1,943
1,189
1,359
5,774
V0
V1
V2
V3
V4
C Output and incomplete accounting
LP/P (n=973)
B/LB (n=1,187)
0
20
40
60
80
100
Percentage of all source-labelled anchors
V0
V1–V3
V4
Incomplete
D LP/P-directed pair at V2
Both conventional
One of each
Both tree
0
9
16
0.0
2.5
5.0
7.5
10.0
12.5
15.0
17.5
Anchors at exact 2:2 disagreement
