# Supplementary material for: HBOCpred: An Ensemble Framework for Prioritisation of Germline Missense Variants in Hereditary Breast and Ovarian Cancer
Source: Int J Mol Sci. 2026 Oct 5;27(19):8865. doi: 10.3390/ijms27198865 (PMC13665518; doi:10.3390/ijms27198865)
Supplement: Supplementary file 1 [file ijms-27-08865-s001.zip › HBOCpred_Reproducibility.zip!/HBOCpred/figures/Figure4_per_gene.pdf]

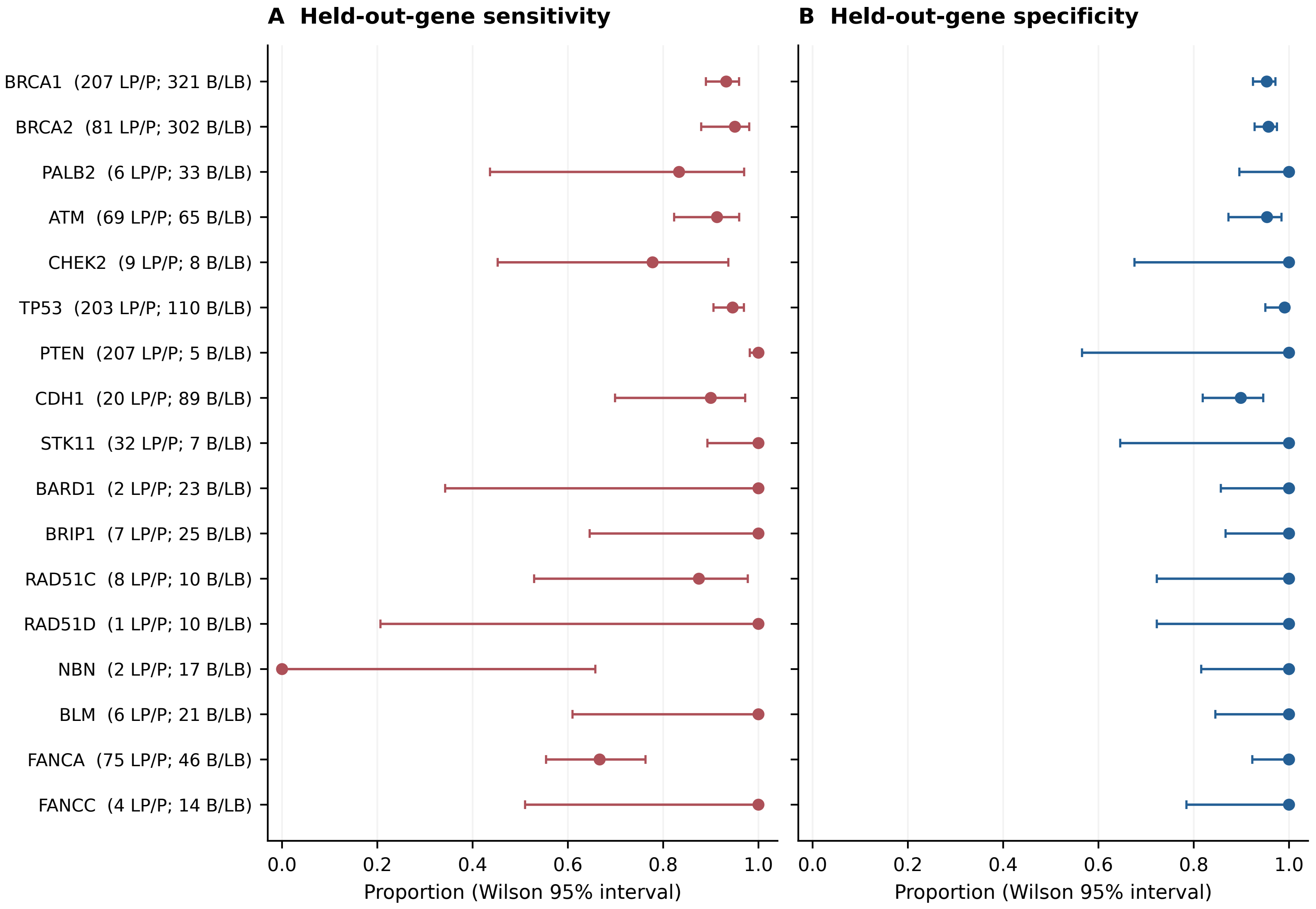

A Held-out-gene sensitivity
B Held-out-gene specificity
BRCA1 (207 LP/P; 321 B/LB)
BRCA2 (81 LP/P; 302 B/LB)
PALB2 (6 LP/P; 33 B/LB)
ATM (69 LP/P; 65 B/LB)
CHEK2 (9 LP/P; 8 B/LB)
TP53 (203 LP/P; 110 B/LB)
PTEN (207 LP/P; 5 B/LB)
CDH1 (20 LP/P; 89 B/LB)
STK11 (32 LP/P; 7 B/LB)
BARD1 (2 LP/P; 23 B/LB)
BRIP1 (7 LP/P; 25 B/LB)
RAD51C (8 LP/P; 10 B/LB)
RAD51D (1 LP/P; 10 B/LB)
NBN (2 LP/P; 17 B/LB)
BLM (6 LP/P; 21 B/LB)
FANCA (75 LP/P; 46 B/LB)
FANCC (4 LP/P; 14 B/LB)
0.0
0.2
0.4
0.6
0.8
1.0
Proportion (Wilson 95% interval)
Proportion (Wilson 95% interval)
