# Supplementary material for: HBOCpred: An Ensemble Framework for Prioritisation of Germline Missense Variants in Hereditary Breast and Ovarian Cancer
Source: Int J Mol Sci. 2026 Oct 5;27(19):8865. doi: 10.3390/ijms27198865 (PMC13665518; doi:10.3390/ijms27198865)
Supplement: Supplementary file 1 [file ijms-27-08865-s001.zip › HBOCpred_Reproducibility.zip!/HBOCpred/figures/Figure5_comparators.pdf]

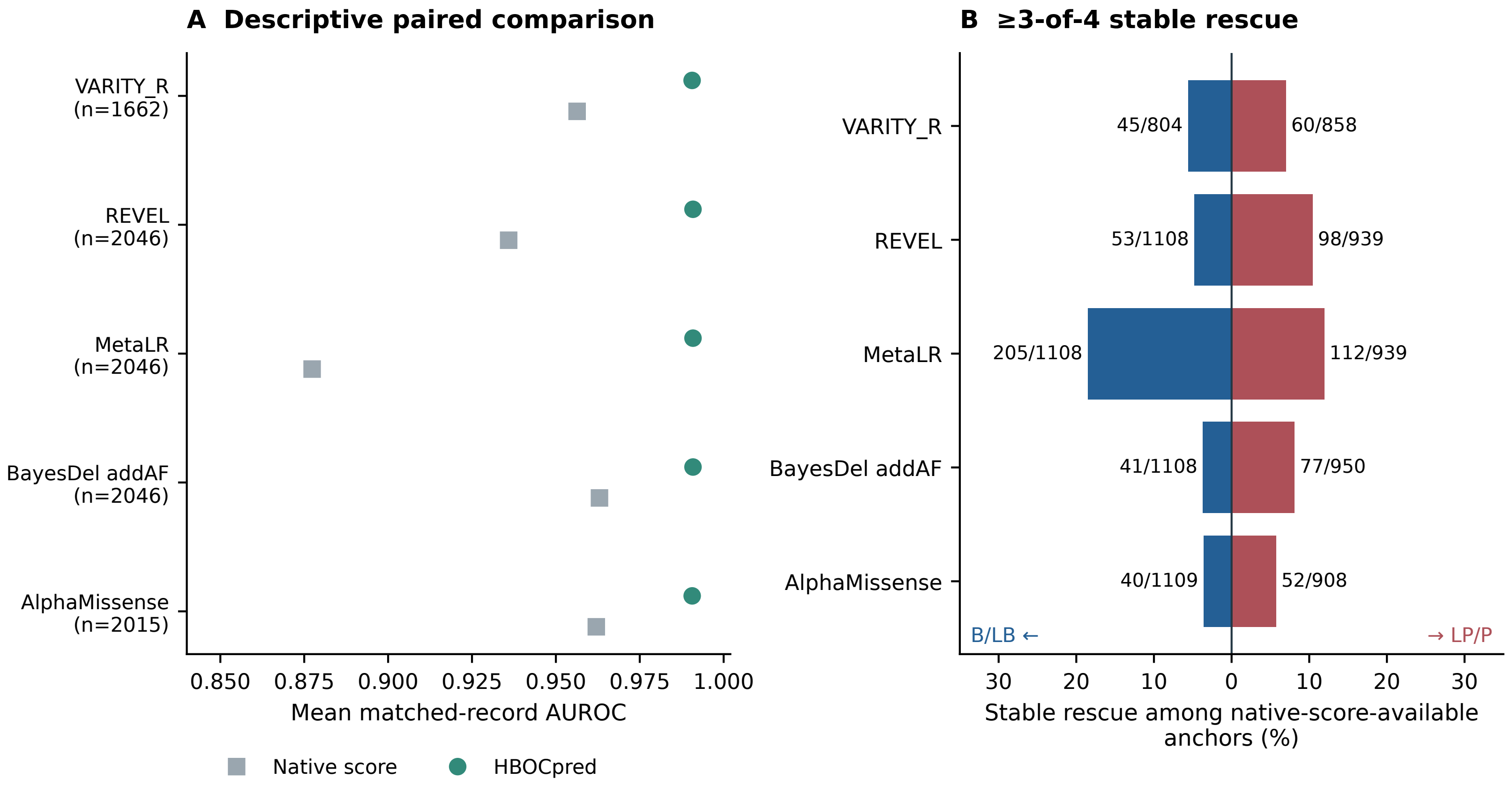

A Descriptive paired comparison
VARITY_R (n=1662)
REVEL (n=2046)
MetaLR (n=2046)
BayesDel addAF (n=2046)
AlphaMissense (n=2015)
0.850
0.875
0.900
0.925
0.950
0.975
1.000
Mean matched-record AUROC
Native score
HBOCpred
B ≥3-of-4 stable rescue
VARITY_R
REVEL
MetaLR
BayesDel addAF
AlphaMissense
45/804
60/858
53/1108
98/939
205/1108
112/939
41/1108
77/950
40/1109
52/908
B/LB ←
→ LP/P
30
20
10
0
10
20
30
Stable rescue among native-score-available anchors (%)
