# Supplementary material for: HBOCpred: An Ensemble Framework for Prioritisation of Germline Missense Variants in Hereditary Breast and Ovarian Cancer
Source: Int J Mol Sci. 2026 Oct 5;27(19):8865. doi: 10.3390/ijms27198865 (PMC13665518; doi:10.3390/ijms27198865)
Supplement: Supplementary file 1 [file ijms-27-08865-s001.zip › HBOCpred_Reproducibility.zip!/HBOCpred/figures/Figure6_review_status.pdf]

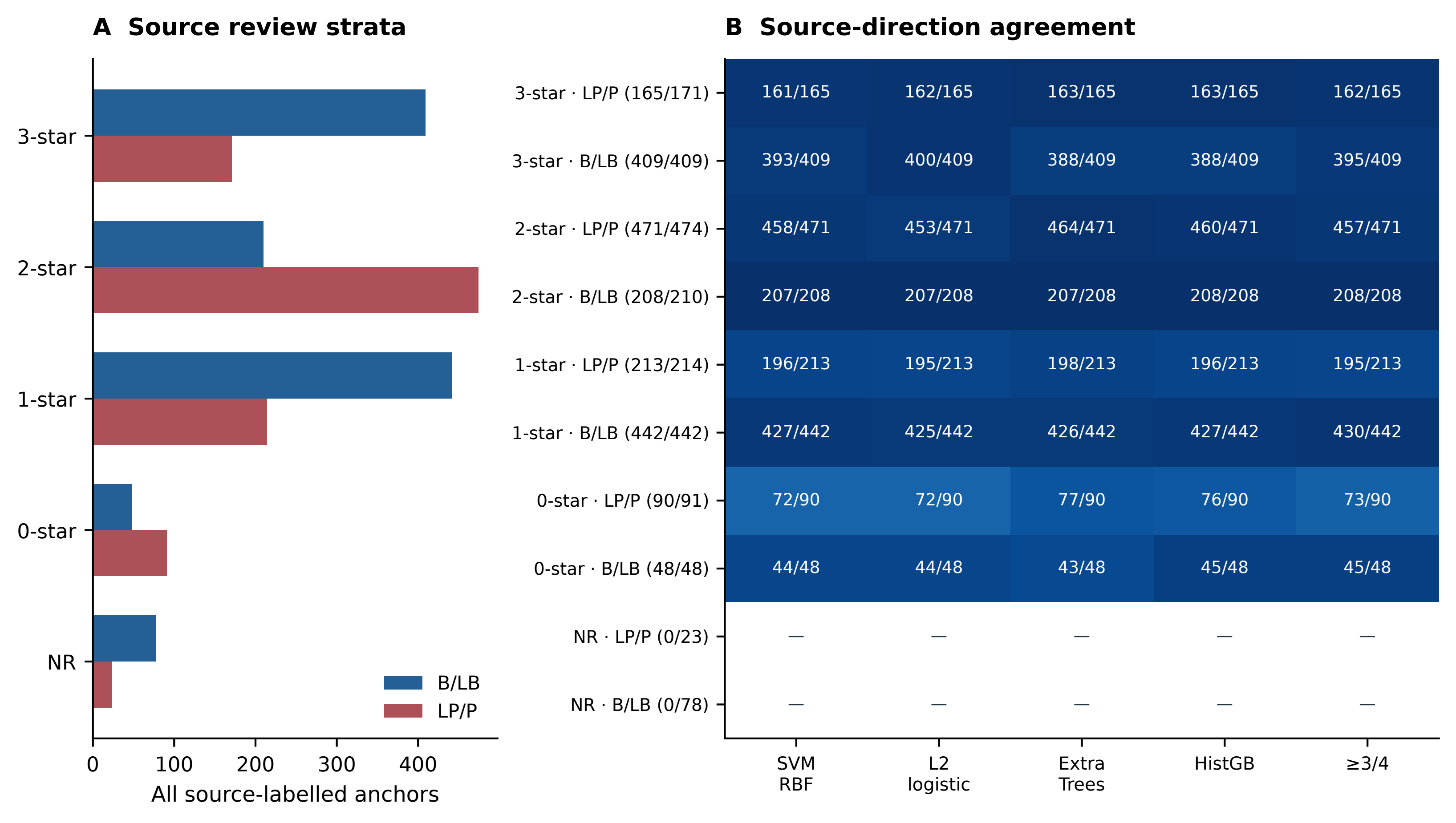
A Source review strata
3-star
2-star
1-star
0-star
NR
B/LB
LP/P
0
100
200
300
400
All source-labelled anchors
B Source-direction agreement
3-star · LP/P (165/171)
3-star · B/LB (409/409)
2-star · LP/P (471/474)
2-star · B/LB (208/210)
1-star · LP/P (213/214)
1-star · B/LB (442/442)
0-star · LP/P (90/91)
0-star · B/LB (48/48)
NR · LP/P (0/23)
NR · B/LB (0/78)
161/165 162/165 163/165 163/165 162/165
393/409 400/409 388/409 388/409 395/409
458/471 453/471 464/471 460/471 457/471
207/208 207/208 207/208 208/208 208/208
196/213 195/213 198/213 196/213 195/213
427/442 425/442 426/442 427/442 430/442
72/90 72/90 77/90 76/90 73/90
44/48 44/48 43/48 45/48 45/48
— — — — —
— — — — —
SVM RBF
L2 logistic
Extra Trees
HistGB
≥3/4
