# Supplementary material for: HBOCpred: An Ensemble Framework for Prioritisation of Germline Missense Variants in Hereditary Breast and Ovarian Cancer
Source: Int J Mol Sci. 2026 Oct 5;27(19):8865. doi: 10.3390/ijms27198865 (PMC13665518; doi:10.3390/ijms27198865)
Supplement: Supplementary file 1 [file ijms-27-08865-s001.zip › HBOCpred_Reproducibility.zip!/HBOCpred/figures/Figure7_shift_gene_signal.pdf]

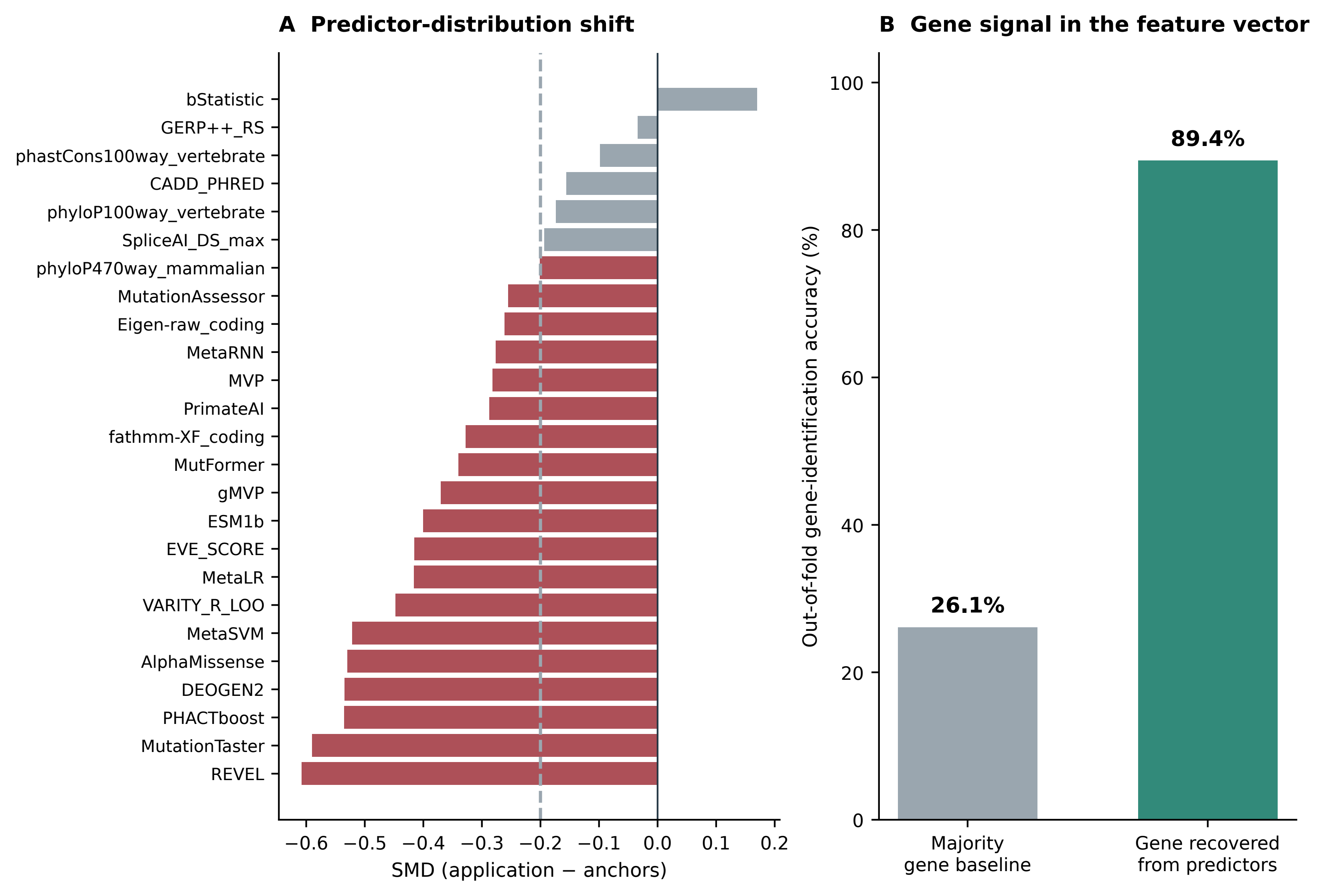
A Predictor-distribution shift
bStatistic
GERP++_RS
phastCons100way_vertebrate
CADD_PHRED
phyloP100way_vertebrate
SpliceAI_DS_max
phyloP470way_mammalian
MutationAssessor
Eigen-raw_coding
MetaRNN
MVP
PrimateAI
fathmm-XF_coding
MutFormer
gMVP
ESM1b
EVE_SCORE
MetaLR
VARITY_R_LOO
MetaSVM
AlphaMissense
DEOGEN2
PHACTboost
MutationTaster
REVEL
−0.6 −0.5 −0.4 −0.3 −0.2 −0.1 0.0 0.1 0.2
SMD (application − anchors)
B Gene signal in the feature vector
Out-of-fold gene-identification accuracy (%)
0 20 40 60 80 100
26.1%
89.4%
Majority gene baseline
Gene recovered from predictors
