# Supplementary material for: HBOCpred: An Ensemble Framework for Prioritisation of Germline Missense Variants in Hereditary Breast and Ovarian Cancer
Source: Int J Mol Sci. 2026 Oct 5;27(19):8865. doi: 10.3390/ijms27198865 (PMC13665518; doi:10.3390/ijms27198865)
Supplement: Supplementary file 1 [file ijms-27-08865-s001.zip › HBOCpred_Reproducibility.zip!/HBOCpred/figures/FigureS1_gnomAD_eligibility.pdf]

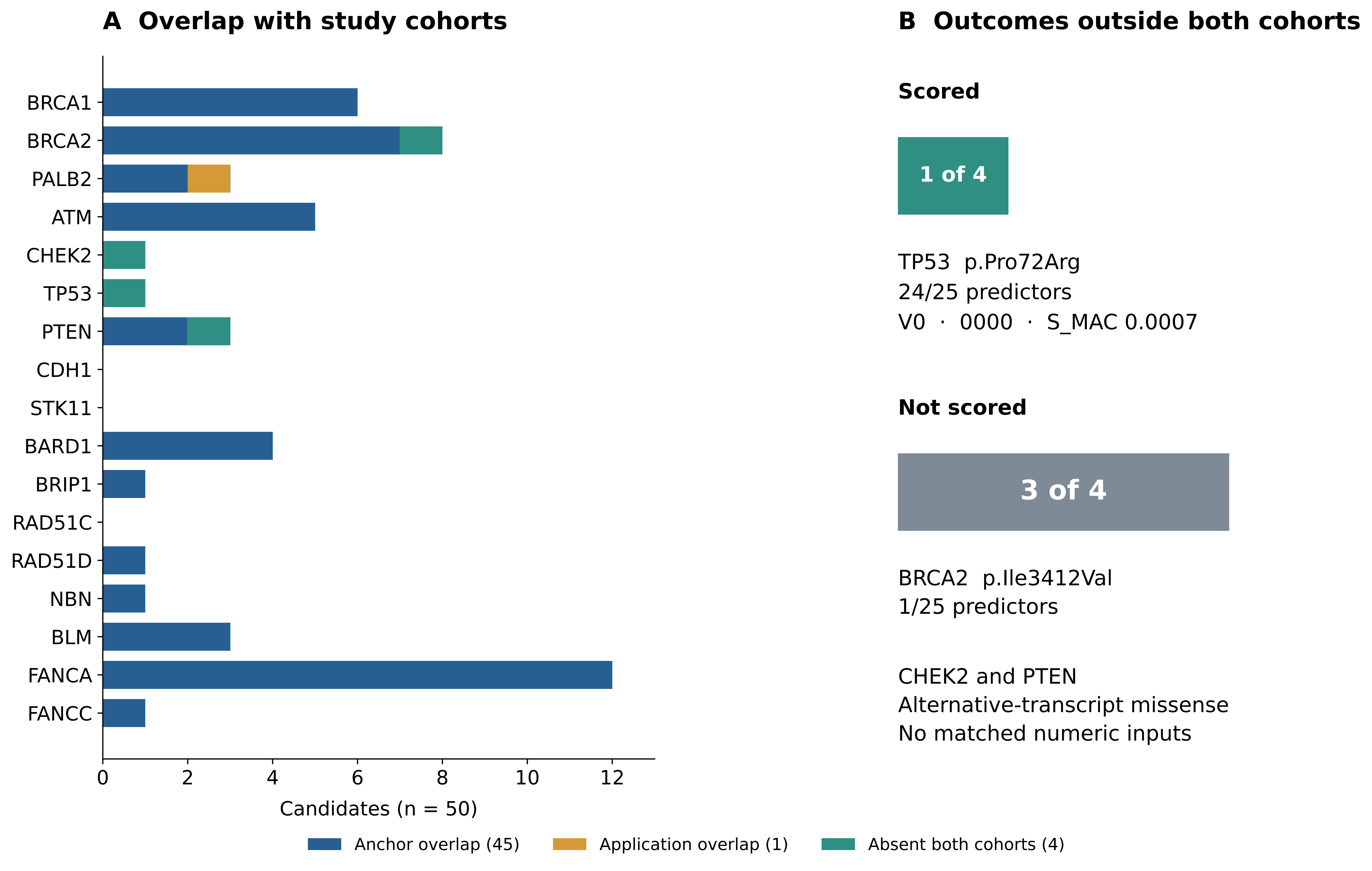

A Overlap with study cohorts
BRCA1
BRCA2
PALB2
ATM
CHEK2
TP53
PTEN
CDH1
STK11
BARD1
BRIP1
RAD51C
RAD51D
NBN
BLM
FANCA
FANCC
0
2
4
6
8
10
12
Candidates (n = 50)
Anchor overlap (45)
Application overlap (1)
Absent both cohorts (4)
B Outcomes outside both cohorts
Scored
1 of 4
TP53 p.Pro72Arg
24/25 predictors
V0 · 0000 · S_MAC 0.0007
Not scored
3 of 4
BRCA2 p.Ile3412Val
1/25 predictors
CHEK2 and PTEN
Alternative-transcript missense
No matched numeric inputs
